# From Innovation to Underutilization: Why Chest Wall Perforator Flaps Have Not Achieved Widespread Adoption—Insights from the Literature and a High-Volume Single-Center Cohort

**DOI:** 10.3390/cancers18193100

**Published:** 2026-09-23

**Authors:** Theodoros Kontoulis, Panagiota Koutra

**Affiliations:** 1 Genesis Hospital, 55535 Thessaloniki, Greece; 2 2nd Surgical Department, Aristotle University of Thessaloniki, 54124 Thessaloniki, Greece

**Keywords:** breast-conserving surgery, chest wall perforator flap, oncoplastic breast surgery, partial breast reconstruction

## Abstract

**Background/Objectives**: Chest wall perforator flaps (CWPF) have emerged as versatile muscle-sparing volume replacement techniques in oncoplastic breast surgery (OBS), facilitating breast conservation in selected patients with favorable tumor-to-breast ratio. However, their adoption into routine practice remains limited. The aim of this study was to explore the factors contributing to the underutilization of CWPF in OBS through a narrative review of the current literature along with insights from a high-volume single-center experience in Greece. **Materials and Methods**: A comprehensive search of PubMed/MEDLINE and Scopus (2004–2026) was performed using predefined keywords related to CWPF, partial breast reconstruction (PBR), breast-conserving surgery (BCS) and OBS. Eligible studies addressing surgical outcomes, patient selection, technical variations, patient satisfaction and barriers to implementation were analyzed qualitatively, due to heterogeneity of study designs and outcome reporting. The institutional cohort included patients undergoing CWPF-based PBR following BCS between May 2018 and December 2025, with data extracted from a prospectively maintained institutional database and analyzed descriptively. **Results**: The literature confirmed CWPF as safe and feasible with low complication rates, reassuring oncological outcomes and satisfactory aesthetic results, with lateral intercostal artery perforator (LICAP) being the most commonly reported technique. However, significant variability in patient selection, technical approach and outcome reporting was observed, alongside limited long-term data. Overall, underutilization appears to be primarily driven by insufficient and non-standardized training with a steep learning curve, limited exposure and a persistent gap between perceived importance and routine clinical availability. In our institution, of the 107 patients who were selected for local flaps, 81 patients underwent CWPF reconstruction and were eligible for the study. LICAP was the most frequently used flap (46.9%), followed by lateral thoracic artery perforator (LTAP), anterior intercostal artery perforator (AICAP) and medial intercostal artery perforator (MICAP) variants. Negative margins were achieved in all cases, with a low complication rate (3.7%) and one local recurrence. No re-excision was required. **Conclusions**: CWPF appear to remain underutilized in routine oncoplastic practice, as indicated by the available literature, reflecting a potential gap between their perceived clinical value and real-world implementation. This underscores the need for structured training and improved dissemination to support broader adoption.

## Simple Summary

Chest wall perforator flaps (CWPF) have emerged as an important option for partial breast reconstruction, allowing breast conservation in patients who might otherwise require mastectomy or more extensive reconstructive procedures. Despite growing evidence supporting their safety, low complication rates and favorable cosmetic outcomes, their use remains limited in routine clinical practice. This study combines a review of the published literature with a large single-center institutional experience to evaluate clinical outcomes and explore factors influencing adoption of these techniques. The findings demonstrate that CWPF are a reliable reconstructive option associated with favorable surgical, oncological and aesthetic outcomes. However, substantial variation exists in patient selection, surgical practice, outcome reporting and training exposure. The study highlights the gap between evidence and implementation and suggests that wider adoption may be facilitated through standardized training pathways, consensus-based guidelines and improved reporting of outcomes.

## 1. Introduction

Breast-conserving surgery (BCS) combined with radiotherapy (RT) is well established as the standard of care for the management of early-stage breast cancer [1,2,3]. Landmark randomized trials have demonstrated equivalent survival outcomes between BCS and mastectomy, while subsequent observational studies suggested improved cosmetic results, patient satisfaction and quality of life (QoL), following breast conservation [1,3,4]. The evolution of oncoplastic breast surgery (OBS) has further expanded the indications for breast conservation by integrating reconstructive techniques with oncological resection, allowing wider excisions without compromising aesthetic outcome [1,2,3,4,5,6].

Oncoplastic techniques are broadly classified into volume displacement procedures, such as glandular reshaping and therapeutic mammoplasty and volume replacement techniques with utilization of autologous tissue outside of the breast to restore breast contour following larger resections [2,4,5]. Although volume displacement techniques are widely used, particularly in patients with medium-to-large or ptotic breasts, they may not be suitable in women with small, non-ptotic breasts or in cases with a high tumor-to-breast volume ratio, where excision of more than 20% of the breast tissue may result in significant deformity and unacceptable cosmetic outcomes [1,2,5]. In contrast, volume replacement techniques enable partial breast reconstruction (PBR) following approximately 20–50% volume loss by replacing the defect with tissue from outside the breast, thereby facilitating breast conservation while maintaining breast shape and symmetry [1,3,5,6].

Historically, latissimus dorsi (LD)-based techniques represented a traditional volume-replacement approach in BCS, with multiple reports describing their use for reconstruction of partial breast defects and extension of breast conservation [1,7,8,9,10,11]. However, despite its reconstructive reliability, LD flap harvest is associated with donor-site morbidity, postoperative pain, functional impairment and potential shoulder dysfunction due to muscle sacrifice [1,2,6]. Increasing understanding of chest wall perforator vascular anatomy subsequently led to the development of muscle-sparing reconstructive alternatives in the form of chest wall perforator flaps (CWPFs) [2]. CWPFs are pedicled adipocutaneous flaps based on perforating vessels arising from the lateral thoracic, thoracodorsal, or intercostal vascular systems, enabling transfer of tissue from the lateral, inferior, or anterior chest wall without sacrificing underlying musculature [3,5]. Intercostal perforators arise from a vascular network between the posterior intercostal and internal mammary circulations and provide dorsal, lateral and anterior perforators, forming the anatomical basis for the different CWPF designs [12].

Hamdi et al. [13] first described the use of thoracodorsal artery perforator (TDAP) and intercostal artery perforator (ICAP) flaps for breast reconstruction in 2004, demonstrating the feasibility and safety of perforator-based PBR [1,2,14]. Subsequently, additional CWPF variants were introduced, including the lateral intercostal artery perforator (LICAP), anterior intercostal artery perforator (AICAP) and medial intercostal artery perforator (MICAP) flaps. McCulley et al. [15] later described the lateral thoracic artery perforator (LTAP) flap as an additional option for reconstruction of lateral breast defects [5,6,14]. In parallel, technical refinements such as those described by Meybodi et al. [16] improved the original LICAP technique by simplifying patient positioning and optimizing incision design to enhance scar concealment and operative efficiency [17]. The choice among these flaps is primarily guided by tumor location and the anticipated defect, but is further individualized according to defect volume, skin requirements, flap reach, breast size, donor-site tissue availability, perforator anatomy and the planned axillary procedure [1,2,3,10,13,18]. These techniques have gained increasing attention as versatile reconstructive options in OBS and have expanded the indications for PBR, particularly in patients with small-to-moderate-sized breasts, larger tumors and defects unsuitable for volume displacement approaches. Moreover, CWPF also contribute to mastectomy avoidance, representing a tissue-preserving, implant-free option within oncoplastic breast conservation in selected patients with a high tumor-to-breast volume ratio [19].

Although CWPFs for PBR were first described nearly two decades ago, their adoption into routine clinical practice has remained relatively limited [3,6]. The PARTBRECON study, a UK multicenter retrospective cohort study by Agrawal et al. [6], demonstrated acceptable short- and medium-term surgical outcomes following CWPF-based PBR, while multicenter study by Karakatsanis et al. [3] highlighted the growing international interest in CWPF techniques and their potential role in reducing mastectomy rates in selected patients. Furthermore, an international survey study by Karakatsanis et al. [4] demonstrated that although most breast surgeons considered CWPF reconstruction an important oncoplastic technique, only a minority routinely offered these procedures in clinical practice, highlighting persistent gaps in training and technical standardization. Nevertheless, recent systematic reviews continue to demonstrate substantial heterogeneity within the existing literature, including variation in patient selection, flap choice, surgical techniques, outcome assessment and reporting of patient-reported outcomes [5]. Existing studies have primarily focused on surgical outcomes or surgeon perceptions, while the factors contributing to the limited dissemination and implementation of CWPF techniques in contemporary breast surgical practice remain insufficiently explored [1,2,3,4,5,6,8,9,10,11,12,13,15,16,17,18,20,21,22,23,24,25,26,27,28,29,30,31,32,33,34,35,36,37,38,39,40]. In particular, there is limited real-world evidence integrating clinical outcomes with practical insights from high-volume centers routinely performing these procedures.

Therefore, the aim of the present study was to explore the factors contributing to the underutilization of CWPF in contemporary OBS through a narrative review of the current literature combined with insights from a high-volume single-center experience in a country with low uptake. Secondary aims included the evaluation of surgical outcomes within our institutional cohort and comparison of these findings with currently available evidence to identify potential barriers to the wider implementation and dissemination of CWPF techniques.

## 2. Materials and Methods

### 2.1. Study Characteristic

This study was designed as a narrative review of the current literature regarding the use of CWPF in OBS, combined with a retrospective analysis of a single-center experience.

### 2.2. Literature Review Methodology

A literature search was performed using the PubMed/MEDLINE and Scopus databases to identify relevant studies evaluating CWPF techniques in the setting of BCS and PBR. The search strategy included “chest wall perforator flap”, “LICAP”, “AICAP”, “MICAP”, “LTAP”, “TDAP”, “partial breast reconstruction”, “breast conserving surgery” and “oncoplastic breast surgery”. The literature published between April 2004 and March 2026 was considered eligible for review. Priority was given to original research articles, multicenter cohort studies, systematic reviews and survey-based studies assessing surgical outcomes, patient selection, oncological safety, aesthetic outcomes, technical variations, training patterns and implementation barriers associated with CWPF techniques. Studies focusing exclusively on volume displacement techniques, implant-based reconstruction (IBR), total breast reconstruction following mastectomy, or non-perforator flap techniques were excluded. Exclusion criteria also included isolated case reports, editorials, expert opinions, conference abstract and reconstructions performed for non-oncologic indications.

Due to the heterogeneity of the available literature in terms of study design, outcome reporting, follow-up duration and patient populations, a narrative synthesis approach was adopted. No systematic review methodology or meta-analysis was conducted. Two reviewers independently assessed the identified literature for relevance and eligibility, and any disagreements were resolved by consensus. Selected studies were analyzed qualitatively, with emphasis on surgical outcomes, patient selection, patterns of clinical adoption and challenges associated with the integration of CWPF techniques within contemporary OBS. No quantitative pooling of outcomes was undertaken. In addition, insights from a high-volume single-center experience were incorporated to provide real-world context and to support the discussion of adoption barriers.

### 2.3. Patients: Inclusion–Exclusion Criteria

A retrospective observational study was conducted including patients who underwent PBR with CWPF at our institution Genesis Hospital—Imithea Medical Group between May 2018 and December 2025.

Eligible patients included those with histologically confirmed breast cancer, including invasive carcinoma and ductal carcinoma in situ (DCIS), who underwent BCS with immediate reconstruction using CWPF techniques and had complete perioperative and follow-up data available. Patient selection for CWPF reconstruction was based on multidisciplinary evaluation and shared decision-making. Indications included multifocal or multicentric disease, large tumors or high tumor-to-breast volume ratio and moderate-sized tumors in patients with small, non-ptotic breasts where standard volume displacement techniques were deemed insufficient (Figure 1 and Figure 2). For the purposes of this study, eligible patients had tumors measuring 2–5 cm, an estimated tumor-to-breast volume ratio of 20–30% and an anticipated excision volume of approximately 20–30% of the breast volume. Patients anticipated to require excision of more than 30% of breast volume were excluded. Tumor location and expected defect geometry (including lower pole defects and upper inner quadrant lesions) were also key determinants of reconstructive planning. Patients undergoing mastectomy with or without IBR, those requiring non-perforator flap reconstruction techniques and patients with incomplete clinical records or inadequate follow-up were excluded from the analysis.

### 2.4. Data Collection

Clinical data, including demographic characteristics, tumor pathology, surgical details, adjuvant treatment and follow-up information, were retrieved from institutional medical records. Data were entered into a dedicated database and stored in a pseudo-anonymized format using Microsoft Excel.

### 2.5. Surgical Technique

All procedures were performed by a single breast surgeon as part of a standardized institutional oncoplastic protocol. Breast-conserving oncological resection and CWPF reconstruction were performed as a single-stage procedure. Preoperatively, breast cup size was recorded, and the dominant perforator vessels were mapped using a handheld Doppler ultrasound probe (8 MHz). Tumor location, anticipated defect and flap design were marked in the preoperative setting. Multiple perforators were routinely identified and marked to allow intraoperative selection of the most appropriate perforator(s), depending on defect size and flap mobility requirements, as shown in Figure 3. In selected cases, more than one perforator was incorporated to enhance flap perfusion and volume. Secondary perforators were preserved, when necessary, particularly in larger reconstructions (Figure 4).

Our flap-selection protocol is defect- and patient-specific and is summarized in Figure 5. Flap selection was guided primarily by tumor location and the expected post-excisional defect, according to the McCulley–McMillan zones, and was subsequently individualized according to breast size, required replacement volume, skin requirements, donor-site tissue availability, perforator anatomy and the planned axillary procedure. Potentially suitable perforators were identified preoperatively using Doppler ultrasonography.

For lateral and lower-outer defects, LICAP flaps were generally the preferred option. LTAP flaps were considered for upper and lateral defects when a reliable lateral thoracic perforator was identified and was not expected to be compromised by axillary surgery. AICAP flaps were selected for inferior and lower-central defects, whereas MICAP flaps were used primarily for medial or lower-medial defects. For larger or extended lateral defects, a combined LICAP–LTAP reconstruction could be considered when additional volume replacement was required.

Following tumor excision, the final defect dimensions and the integrity of the planned perforator were reassessed. When the initially selected perforator was injured, compromised or unsuitable for the final defect, an alternative preoperatively mapped perforator or flap design was used where feasible.

Axillary management, sentinel lymph node biopsy (SLNB) or axillary lymph node dissection (ALND), was performed according to standard oncological indications and, when feasible, through the same incision used for wide local excision (WLE) or flap harvest. Care was taken to preserve the lateral thoracic vascular system, especially in cases involving LTAP flaps.

WLE was performed through the planned flap incision in the majority of cases. Following tumor excision, CWPF elevation was carried out in a lateral-to-medial direction. Reconstruction was performed using LICAP, LTAP, MICAP or AICAP flaps, selected according to tumor location and defect geometry (Figure 5). Flaps were designed as pedicled perforator-based adipocutaneous tissue transfers and were inset either by rotation (clockwise or counterclockwise) using the propeller technique, or by turnover with 180° folding around the perforator. Muscle-sparing dissection was performed in all cases, preserving the underlying LD and serratus anterior muscles. Flaps were rotated or transposed carefully to avoid torsion or kinking of the vascular pedicle and secured with absorbable Vicryl sutures.

Intraoperative frozen section analysis was routinely performed to ensure negative surgical margins, with final histopathological assessment confirming oncological adequacy according to standard criteria. In cases of involved margins, re-excision of the cavity was performed when required. All cases were discussed in a multidisciplinary tumor board to determine adjuvant treatment planning.

Postoperative follow-up included clinical assessment and structured reviews at 6 months and 1 year, with continued follow-up after completion of adjuvant RT. Complications and aesthetic outcomes were assessed through clinical examination and photographic documentation at scheduled follow-up visits. Surgeons evaluated aesthetic outcomes by comparison of preoperative and postoperative clinical findings and images.

### 2.6. Study Outcomes

The primary outcome was overall surgical safety and feasibility of CWPF reconstruction, assessed through postoperative complication rates and oncological adequacy, including margin status, re-excision rates and local recurrence during follow-up.

Secondary outcomes included early postoperative complications occurring within 30 days, such as seroma, surgical site infection (SSI), wound related complications (including wound dehiscence and delayed wound healing), partial or total flap necrosis, hematoma, lymphoedema and donor-site morbidity. Fat necrosis was also recorded during follow-up. Complications were documented when they required medical or surgical intervention, including unplanned outpatient visits, readmission, or reoperation.

Additional secondary outcomes regarding perioperative and postoperative characteristics were operative time for flap harvest, drain usage, duration of hospital stay, readmission and reoperation. Where applicable, aesthetic outcomes were also reviewed as part of routine postoperative clinical and photographic assessment.

The results of this study were compared to results reported in the literature.

In addition, practical observations related to the integration of CWPF techniques into routine clinical practice were considered during data interpretation to support discussion regarding their implementation in contemporary OBS.

### 2.7. Ethical Considerations

The study was conducted in accordance with institutional ethical standards and in accordance with the Declaration of Helsinki. Institutional approval was obtained by the Institutional Review Board of Genesis Hospital—Imithea Medical Group (GHIMG001-15-10-2018, 15 October 2018). Given its retrospective design, formal informed consent was not required for data analysis. All patient data were anonymized prior to analysis to ensure confidentiality. Written informed consent was obtained from all patients for clinical photography and for the use of images where applicable.

### 2.8. Statistical Analysis

Descriptive statistics were used to summarize patient characteristics and outcomes. Continuous variables were reported as mean values with ranges, while categorical variables were expressed as frequencies and percentages.

### 2.9. GenAI Use

Generative artificial intelligence tools were used to support manuscript structuring, drafting and refinement of selected sections of text, and development of illustrative figures. Specifically, ChatGPT (OpenAI; Version 1.2026.153) was used during manuscript preparation. The authors were fully responsible for the literature search, data collection, analysis, interpretation and final manuscript content.

## 3. Results

### 3.1. Literature Findings

The literature review included 31 studies, the majority of which were retrospective single-center cohort studies. Only three studies were multicenter in design [3,6,12], while a smaller number were prospective series [1,11,12,15,16,24,27,28,29,32,34]. The geographical distribution of studies was heterogeneous, although most originated from centers in the United Kingdom [3,6,11,12,15,20,21,22,23,26,27,38]. Study characteristics are summarized in Table 1.

Reporting quality varied considerably across the literature. Population characteristics, operative details and outcome measures were frequently incompletely reported, with substantial amounts of missing data across studies. In addition, definitions of clinical outcomes and complications were not standardized, limiting direct comparison between cohorts.

Breast size was reported in approximately half of the included studies, with most cohorts comprising patients with cup sizes ranging from A to D [1,6,9,11,20,22,23,26,27,30,33,34,35,37,38]. Breast ptosis was documented in only two studies [30,34]. LICAP was the most frequently utilized CWPF technique across the literature. Follow-up duration was generally limited and inconsistently reported [1,3,6,8,9,10,11,12,16,17,22,23,25,26,27,29,31,32,33,35,36,37]. Losses to follow-up were poorly documented in most studies, apart from Agrawal et al., who provided detailed information regarding patient attrition [1].

Reported complication rates were generally low. Seroma, wound dehiscence and fat necrosis were the most commonly described complications, although definitions varied substantially between studies. Margin status was reported in 26 studies [1,3,6,8,9,11,12,16,17,20,21,22,23,24,25,26,27,29,30,32,33,34,35,36,37,38]. Re-excision rates ranged from 0% to 18%, while only nine studies specifically reported completion mastectomy rates to achieve clear margins [1,3,12,20,23,26,33,38]. Local recurrence was not consistently reported and ranged from 0.65% to 6.6% among studies providing these data [1,4,6,8,10,11,12,17,20,24,26,27,30,33,34,36,38]. However, interpretation of these oncological outcomes is limited by heterogeneous and frequently insufficiently reported follow-up durations across studies. Distant recurrence was reported in 13 studies and ranged from 0% to 10.5% [1,4,6,10,11,12,20,26,27,30,33,34,36,38].

Major oncological outcomes were infrequently reported. Overall survival, disease-free survival and other long-term oncological endpoints were described in only four studies (12.9% of the included literature), reflecting the limited availability of long-term oncological data [20,33,36,38].

Patient-reported cosmetic outcomes (PROMs) were evaluated in nine studies [8,10,17,20,30,31,32,35,36]. Four validated PROM instruments were identified, including the Breast-Q questionnaire [20,30,35,36], the Structured Data Capture 1 (SDC-1) PROM tool [31], the 3-Month Mastectomy Questionnaire [32] and the Kyungpook National University Hospital Breast Satisfaction Survey [8,17]. Across studies reporting PROMs [8,10,17,20,30,31,32,35,36], most patients expressed high levels of satisfaction with cosmetic outcomes, with the majority rating breast appearance as good or excellent.

Only one study reported the routine use of indocyanine green (ICG) angiography for flap perfusion assessment [1]. Roy et al. described a two-stage reconstructive approach, whereas most studies employed a single-stage reconstruction strategy [27]. The need for contralateral symmetrization procedures was reported in only two studies, with rates of 1.2% and 4.7%, respectively [6,37]. Furthermore, only one study specifically reported radiotherapy-related outcomes following CWPF reconstruction [35].

Three systematic reviews were identified. The most recent review by Yadav et al. [5] (2026) included 52 studies comprising 2591 patients. Pujji et al. [18] (2021) analyzed 11 studies involving 432 patients, while Nava et al. [2] (2025) performed the only meta-analysis to date, including 17 studies and 1893 patients. LICAP was reported as the most commonly utilized flap across all three reviews. Characteristics of the systematic reviews are summarized in Table 2.

Notably, only two studies specifically explored factors influencing CWPF adoption and implementation in routine clinical practice, highlighting the relative scarcity of literature addressing barriers to dissemination and underutilization [4,34].

### 3.2. Institutional Cohort

#### 3.2.1. Baseline Characteristics

During the 7-year period (May 2018 and December 2025), a total of 81 patients underwent CWPF reconstruction for PBR at our institution. To our knowledge, this represents the largest CWPF single-center reported series in Greece. Among them, 70 (86.4%) patients underwent upfront surgery, while 11 (13.6%) patients received neoadjuvant therapy (NAT) prior to surgery, primarily in patients with TNBC and HER2-positive disease. The median age at the time of surgery was 57 years (range 37–74 years). Most patients had B-cup breasts (51.8%). Overall, 14 (17.3%) patients were diagnosed with DCIS, including two cases with micro-invasive foci (14.3% of DCIS cases), while 67 patients (82.7%) had invasive carcinoma. Multifocal disease was identified in 24 of the invasive carcinoma cases (35.8%). The median pathological tumor size was 31 mm (range 16–55 mm). Patient, tumor and treatment characteristics are summarized in Table 3.

All patients underwent immediate reconstruction following confirmation of negative resection margins by intraoperative frozen section analysis. LICAP was the most frequently performed flap, used in 38 (46.9%) patients. Among these, the majority underwent the classic LICAP technique (Figure 6) as originally described by Hamdi et al. [13], while 17 cases (44.7%) involved the Meybodi modification [16] and 4 cases (10.5%) the crescent modification [39,40,41,42]. LTAP, AICAP and MICAP flaps were performed in 20 (24.7%), 18 (22.2%) and 5 (6.2%) cases, respectively (Table 4) (Figure 7, Figure 8 and Figure 9A). Axillary staging with SLNB was performed in the majority of patients with invasive disease and in selected cases of DCIS where invasive components could not be excluded preoperatively (58/81, 71.6%), while ALND (level I–II) was required in 19 patients (23.5%). Adjuvant RT was administered to all patients. Adjuvant treatment was subsequently determined according to multidisciplinary team recommendations and tumor biology.

#### 3.2.2. Oncological Outcomes and Complications

Negative margins were achieved in all patients both on intraoperative frozen section assessment and final histopathological examination and no patient required re-excision surgery. Postoperative complications occurred in 3 of 81 patients, corresponding to an overall complication rate of approximately 3.7% (Figure 9B,C). Two patients required reoperation. One patient developed a donor-site hematoma requiring surgical evacuation, while another experienced venous flap necrosis resulting in total flap loss, which was managed with debridement followed by reconstruction using a LD myocutaneous flap. Two patients developed wound dehiscence with delayed wound healing, both of which were managed conservatively. No cases of seroma, SSI, lymphoedema, or donor-site necrosis were observed. Fat necrosis during follow-up imaging was identified in six (7.4%) patients.

During follow-up, one patient developed local recurrence two years after the initial procedure. The patient had previously undergone WLE with LICAP reconstruction and ALND, followed by adjuvant chemotherapy and RT for Luminal B pT2N1a breast cancer (tumor size 3 cm, 2/16 positive lymph nodes). Recurrence presented as an 8 mm triple-negative breast cancer (TNBC), and completion mastectomy was performed due to inability to deliver repeat RT (Table 5).

No further surgical intervention was required following final histopathological assessment in the remaining cohort. No patient required contralateral symmetrization surgery.

#### 3.2.3. Perioperative and Postoperative Characteristics

The median duration of flap harvest was 18 min (range 13–25). Surgical drains were used in 51 patients (63%), while 30 patients (37%) were managed without drainage and were discharged on the day of surgery. Most patients (49/81, 60.5%) were discharged the day following surgery after an overnight hospital stay of approximately 12–16 h. Two patients (2.5%) required a prolonged hospital stay of 2 days due to reoperation, one for evacuation of a hematoma and one for management of total flap loss. No unplanned hospital readmissions were recorded during the postoperative period (Table 6).

## 4. Discussion

### 4.1. Principal Findings

In our institutional cohort of 81 CWPF reconstructions, only a low rate of predominantly minor complications (3.7%) was observed, with a single case of local recurrence and no re-excisions required following initial surgery. These outcomes reinforce the reproducibility and oncological safety of the technique when applied within a structured oncoplastic workflow. Consolidating the findings of the literature along with our institutional experience, CWPFs demonstrate low complication rates, excellent oncological safety in terms of margin control and re-excision rates, favorable short- to mid-term outcomes and successful application of CWPF techniques across a broad spectrum of breast defects [1,2,3,5,6,8,9,10,11,12,13,18,20,23,24,26,27,28,29,30,31,32,33,34,35,36,38].

Despite these encouraging clinical outcomes, CWPF reconstruction remains inconsistently adopted in routine practice [4]. The combined evidence from our cohort and the literature [4,34] suggests that this discrepancy is unlikely to be explained by clinical performance alone but rather reflects broader factors including variability in patient selection, limited standardization of indications, technical familiarity and disparities in training and institutional exposure.

### 4.2. Critical Appraisal of the Literature

The findings of this study are consistent with the growing body of evidence supporting CWPF reconstruction as a low-morbidity, muscle-sparing volume replacement technique in OBS [1,2,5,8,9,10,12,13,20,22,23,24,26,27,28,29,30,31,32,33,34,35,36]. Systematic reviews and multicenter cohort studies have consistently demonstrated low complication rates and acceptable oncological outcomes [2,3,5,6,12,18].

Recent pooled analysis by Yadav et al., including 2591 patients, reported overall complication rates of approximately 13%, re-excision rates ranging from 9 to 16% and local recurrence rates of approximately 1–2% [5]. Similarly, the PARTBRECON multicenter study, involving 507 patients, reported complication rates of 12% and re-excision rates of 15.9%, with local recurrence rates around 1% [6]. These re-excision rates should, however, be interpreted in the context of patient selection and variability in intraoperative margin assessment. CWPFs may be selected for relatively large, multifocal or otherwise challenging lesions requiring wider excision, potentially increasing the risk of margin involvement. Furthermore, the use of intraoperative margin assessment, including frozen-section analysis, was inconsistently reported across studies and may have contributed to variability in re-excision rates. Earlier systematic reviews by Pujji et al. and Nava et al. have reported comparable findings, despite some variation in re-excision rates, likely reflecting heterogeneity in follow-up duration, patient selection and outcome reporting [2,18].

Compared with these published data, our institutional cohort demonstrated lower overall complication rates (3.7%) and no re-excisions, with only a single local recurrence observed during follow-up. This difference may partly reflect the relatively small cohort size and the resulting variability of outcome estimates. Nevertheless, it may also be attributable to careful patient selection, surgical standardization and routine intraoperative margin assessment, it further supports the reproducibility of CWPFs in specialized high-volume centers.

When complication profiles are examined in greater detail, most adverse events reported across the literature are minor [1,2,5,8,10,11,12,13,18,20,23,24,26,27,28,30,31,32,33,34,35,36]. In the pooled analysis by Yadav et al., seroma formation was the most frequently reported complication (5%), followed by infection (1.8%), fat necrosis (1.6%), wound dehiscence (1.5%) and total flap loss (0.7%) [5]. Notably, interpretation of seroma remains inconsistent across studies. While many authors report seroma as a postoperative complication, Agrawal et al. argued that donor-site seroma may represent an expected sequel of the procedure rather than a true complication [6]. This highlights the need for greater standardization of complication reporting within the CWPF literature.

Similarly, the PARTBRECON study reported hematoma and wound infection rates of 4.3% and flap loss in 0.6% of patients [6]. In our cohort, hematoma occurred in 1.2% of patients and minor wound dehiscence in 2.5%, possibly due to long scars associated with CWPF, while no seroma was observed. One patient experienced complete flap loss requiring salvage with a LD flap reconstruction. Importantly, none of the complications resulted in deviation from the planned adjuvant treatment pathway. Furthermore, unlike several published series [1,3,12,20,23,26,30,33,38] that reported completion mastectomy rates ranging between approximately 1.5% and 2%, no patient in our cohort required conversion to mastectomy [2,6].

Interestingly, only two studies reported the need for contralateral symmetrization procedures, with rates of 1.2% and 4.7%, respectively [2,6,37]. This finding may reflect one of the aesthetic advantages of CWPF reconstruction, namely the ability to preserve native breast architecture and achieve satisfactory symmetry without routine contralateral surgery. Unlike volume-displacement techniques, which may alter breast shape and frequently require careful balancing procedures in selected patients, CWPFs provide additional autologous volume while maintaining the contralateral breast as a natural reference for symmetry [43,44].

Across the literature, LICAP remains the most frequently utilized flap [1,2,5,6,8,10,11,12,15,18,20,22,23,24,25,26,27,28,29,31,32,33,34,35,36,37,38], followed by AICAP, MICAP, LTAP and TDAP variants, a distribution [5] that is mirrored in our cohort, where LICAP was the predominant technique, used in 46.9% of our patients. This consistency reinforces the anatomical and technical reliability of lateral chest wall perforator-based reconstruction for defects in the lateral and inferior breast quadrants.

### 4.3. Clinical Implications

CWPF reconstruction offers several inherent advantages that support its integration into contemporary OBS. As a muscle-sparing and implant-free volume replacement strategy, it enables restoration of breast volume without compromising shoulder function [1,2,5,18,20,45] or introducing implant-related morbidity [9,26,31]. In contrast to more complex reconstructive approaches, CWPF procedures do not require microsurgical anastomosis [46] or patient repositioning during surgery [16,17,32], contributing to reduced operative complexity and facilitating their incorporation into routine breast-conserving workflows.

From a practical surgical perspective, both our institutional experience and the available literature suggest that CWPF reconstruction can be performed with low morbidity and acceptable resource utilization [34]. Several authors have reported the feasibility of day-case or short-stay surgery [2,3,6,12,23,26,32,45] without routine drainage [3,23,38]. In our cohort, although drains were used in the majority of patients (63%), most patients were managed as overnight admissions with uncomplicated postoperative recovery, reflecting the minimally invasive nature of these procedures.

Beyond their favorable safety profile, CWPFs offer important aesthetic advantages. The technique allows reliable volume replacement while preserving breast contour and symmetry, providing balanced and natural outcomes [2,9,36,47]. Furthermore, scars are typically concealed along the lateral thoracic wall or within the inframammary fold, minimizing visible breast scarring and contributing to favorable cosmetic results [36,37]. These advantages have been consistently reflected in both surgeon-reported and PROMs across the literature [2,4,9,36,37,47].

Importantly, CWPFs are particularly valuable in anatomically challenging situations where conventional volume displacement techniques may be insufficient. Their versatility allows reconstruction of defects across multiple breast quadrants, including lower pole, medial and lateral tumors, as well as moderate-volume resections in small-to-moderate-sized breasts [1,2,13,18]. By providing additional reconstructive options in these settings, CWPFs may expand the indications for BCS and reduce the need for mastectomy in carefully selected patients [3,4].

Moreover, their versatility may also extend beyond partial breast reconstruction. Preliminary experience has demonstrated the feasibility of CWPFs for selected cases requiring whole-breast reconstruction, management of implant-related complications, upper inner quadrant defects and provision of additional skin cover, further broadening the potential reconstructive applications of these flaps [10].

An additional practical advantage of CWPF reconstruction is that it preserves the LD muscle as a future reconstructive option [5,23]. This may be particularly relevant in the event of local recurrence or subsequent reconstructive requirements, providing greater flexibility within the patient’s long-term reconstructive pathway.

These practical and reconstructive advantages may partly explain the growing interest in CWPF reconstruction observed over the last decade.

### 4.4. Institutional Insights and Technical Considerations

Beyond outcome measures, our institutional experience provides practical insights into the implementation of CWPF reconstruction in routine surgical practice, highlighting key technical principles that support reproducibility and safe integration into oncoplastic workflows.

**Stepwise Institutional Adoption of CWPFs: Lessons for Implementation.** Our institutional experience suggests that the adoption of CWPF reconstruction can be facilitated through a structured, stepwise implementation process. Over the 7-year study period, the increasing use of CWPFs in our unit followed comprehensive training in oncoplastic breast surgery, fellowship-based exposure to CWPF techniques and participation in hands-on perforator flap courses. This educational foundation, together with increasing operative experience, progressively improved technical proficiency, clinical decision-making and patient selection.

Implementation was gradual with respect to both case complexity and flap selection. During the initial phase, cases were selected in which the anticipated defect was within the effective reach of the flap and could be reconstructed without excessive flap transposition. LICAP flaps were predominantly used at this stage because of their relatively consistent vascular anatomy and the reliable identification of their perforators. As experience increased, the reconstructive repertoire was expanded to include AICAP and MICAP flaps, while LTAP flaps were incorporated at a later stage.

Preoperative perforator mapping, a flexible location- and defect-based reconstructive algorithm and intraoperative margin assessment with frozen-section analysis supported this progressive implementation. Frozen-section assessment enabled immediate additional excision when indicated, allowing reconstruction to proceed after confirmation of adequate margins. Collectively, dedicated training, progressive case selection, increasing operative experience and structured perioperative planning may provide a practical framework for other institutions seeking to introduce and expand CWPF reconstruction.

**Flap planning and defect assessment** are primarily defect-driven, with decision-making guided by tumor location, resection volume and anticipated soft-tissue requirements. Intraoperative flexibility remains essential, allowing tailoring of flap design to achieve adequate coverage while maintaining vascular reliability. In line with contemporary recommendations, preoperative flap planning may be supported by handheld Doppler ultrasound for identification and mapping of perforator vessels, facilitating more precise flap design and intraoperative dissection.

**Perforator preservation** is a critical step in ensuring flap viability. In larger reconstructions, preservation of more than one perforator is considered whenever feasible, particularly in cases where increased flap perfusion demand is anticipated or where intraoperative assessment suggests borderline vascularity.

**Flap viability assessment and management of vascular compromise** represent important technical considerations during CWPF reconstruction. In our cohort, one patient experienced venous flap congestion resulting in total flap loss, requiring debridement and subsequent reconstruction with a latissimus dorsi myocutaneous flap. Although the exact mechanism of flap failure could not be definitively established, the clinical course was suggestive of venous outflow compromise. This complication highlights the importance of meticulous intraoperative assessment of flap perfusion and early recognition of vascular compromise. Intraoperative evaluation should include an assessment of flap bleeding, skin paddle color and capillary refill, while preservation of an adequate perforator pedicle and avoidance of tension, kinking or compression during tunnelling and inset are essential to minimize the risk of venous outflow impairment. In cases of uncertain perfusion, selective trimming of the distal flap edge or modification of the inset may assist in assessing tissue viability and reducing the risk of ischemic complications. Early recognition of venous congestion remains critical to enable timely intervention and minimize the risk of complete flap loss. This experience reinforces the importance of careful flap selection, intraoperative judgement and systematic monitoring of flap viability as integral components of safe CWPF implementation.

**Flap transfer and inset** are performed via subcutaneous tunnelling towards the defect site, with meticulous attention to minimizing tension, traction or compression of the perforator pedicle and inset. This step is essential to ensure stable perfusion and to reduce the risk of ischemic complications or partial necrosis.

From an oncological perspective, **axillary surgery** can be performed through the same incision whenever anatomically and oncologically appropriate, thereby avoiding additional scars and contributing to a streamlined operative approach.

**Margin assessment is routinely supported by intraoperative frozen section analysis** in our institution, ensuring real-time confirmation of tumor clearance. This strategy may contribute to the absence of re-excision in our cohort and aligns with established oncological principles that prioritize complete excision at the index procedure. It further emphasizes the importance of integrating reconstructive planning with surgical oncology decision-making.

In contrast to several contemporary series [1,36,48], **indocyanine green (ICG) fluorescence angiography is not routinely utilized** in our center for flap perfusion assessment, although its use has been increasingly described in the literature as an adjunct for evaluating flap viability and potentially reducing ischemic complications. Reported flap loss rates in studies utilizing ICG remain low [1,36], suggesting that ICG-guided perfusion assessment may represent a useful tool deserving further evaluation.

Taken together, these observations highlight that beyond technical feasibility, successful CWPF implementation depends on structured operative workflows, careful intraoperative decision-making and multidisciplinary coordination between breast surgeons and the wider oncological team.

### 4.5. Why Are CWPFs Still Underutilized? Factors Contributing to Underutilization of CWPFs

#### 4.5.1. Lack of Standardization

##### Heterogeneity

One of the most consistent findings emerging from the literature is the substantial heterogeneity across published studies on CWPF reconstruction. Significant variability exists in patient selection criteria, flap choice, operative technique, reporting of complications, aesthetic evaluation methods and duration of follow-up. This lack of standardization makes direct comparison between studies challenging and limits the development of robust, evidence-based guidelines regarding optimal indications for CWPF use.

Furthermore, differences in outcome definitions—particularly regarding complications such as seroma [6], fat necrosis and wound morbidity—further contribute to inconsistencies in reported results. As a result, the current evidence base, while broadly supportive of CWPF safety and efficacy, remains methodologically heterogeneous.

##### Aesthetic Outcomes/PROMs

Aesthetic outcomes following CWPF reconstruction are consistently reported as favorable across most published studies [1,3,6,8,12,18,27,30,33,36]. However, meaningful comparison between cohorts is limited by the absence of standardized outcome assessment tools. A wide variety of methods have been used, including surgeon-reported grading systems, non-validated cosmetic scales and selected PROMs such as the Breast-Q [20,30,35,36], Kyungpook Satisfaction Scale [8,17], SDC-1 PROM tool [31] and 3-Month Mastectomy Questionnaire [32].

Despite this variability, available evidence suggests high levels of patient satisfaction, with most studies reporting outcomes in the good-to-excellent range [1,3,6,8,12,18,27,30,33,36]. One of the key advantages of CWPF reconstruction lies in its ability to achieve optimal breast contour with scars concealed along the lateral chest wall or inframammary fold, contributing to improved aesthetic acceptability [2,9,36,37,47].

However, the absence of universally adopted PROMs remains a significant limitation, and there is a clear need for standardized, prospective patient-reported outcome assessment in future CWPF studies.

#### 4.5.2. Challenges in Patient Selection

Patient selection remains one of the least standardized aspects of CWPF reconstruction. Although there is general agreement [1,5,6,8,9,10,11,12,17,20,21,22,23,24,27,29,32,33,34,36,49] that CWPFs are most suitable for patients with small-to-moderate breast size, limited ptosis and relatively high tumor-to-breast volume ratios, formalized selection criteria are lacking in most published series.

The nomogram proposed by Karakatsanis et al. represents one of the few structured attempts to standardize patient selection [4]. This model incorporated breast volumetry and target defect assessment rather than conventional surrogates such as cup size or tumor stage. It identified a highly selected population with a median breast volume of approximately 250 mL, a resection ratio of 28% and unfavorable tumor locations including the lower pole, medial breast, or multifocal/multicentric disease involving multiple quadrants.

This framework highlights an important principle: CWPF reconstruction is most effective when guided by objective anatomical and volumetric parameters rather than subjective surgical judgement alone. Nevertheless, in routine clinical practice, decision-making continues to rely heavily on surgeon experience and intraoperative assessment, resulting in substantial inter-surgeon variability.

The absence of widely accepted and validated selection criteria therefore may represent a key barrier to broader adoption, particularly among surgeons with limited experience in CWPF, who may be less confident in identifying appropriate candidates. Standardization using objective tools may improve consistency in patient selection and facilitate wider dissemination of the technique.

#### 4.5.3. Gaps in the Evidence Base

##### Oncological Outcomes

Although CWPF reconstruction is consistently reported as oncologically safe [1,2,4,8,12,17,18,20], the strength of this conclusion is limited by several methodological factors. Most published studies report relatively short and heterogeneous follow-up periods, typically ranging between 19 and 39 months, which may be insufficient to capture late recurrence patterns in breast cancer [1,3,6,8,9,10,11,12,16,17,22,23,25,26,27,29,31,32,33,35,36,37]. In addition, there is considerable inconsistency in the reporting of tumor biology, systemic therapy and RT regimens, further limiting cross-study comparability.

Taken together, these limitations restrict the ability to draw definitive conclusions regarding long-term oncological outcomes, despite the overall encouraging signal from the available evidence. As demonstrated by Karakatsanis et al. in their comparative analysis of CWPF and mastectomy, CWPF reconstruction enabled breast conservation without increasing complication or re-excision rates, although outcomes should not be directly equated with those observed in other oncoplastic techniques such as therapeutic mammoplasty [3].

##### Radiotherapy

The interaction between CWPF reconstruction and adjuvant RT remains an underexplored but clinically important area. Although RT is an integral component of BCS, relatively few studies have specifically evaluated its effects on CWPF outcomes [35,36]. Consequently, important aspects such as flap volume stability, radiation-induced fibrosis, long-term cosmetic evolution and radiological surveillance remain insufficiently investigated.

Available evidence is generally reassuring. Koppiker et al. reported that the majority of patients receiving adjuvant RT experienced no or only mild-to-moderate toxicity, with no cases of severe radiation-related complications [36]. Similarly, Zeelst et al. reported that 95.2% of patients in their cohort received adjuvant RT, with low related complications, supporting the notion that planned RT does not preclude the use of CWPF reconstruction [35]. However, important questions remain regarding long-term tissue changes, optimal boost delivery and the impact of RT on reconstructed volume and symmetry.

The multicenter PARTBRECON study further highlighted the lack of detailed RT data and emphasized that tumor-bed boost planning following CWPF reconstruction may be technically challenging due to altered tissue anatomy and displacement [6]. This underscores the importance of close multidisciplinary collaboration between breast surgeons and radiation oncologists, particularly during implementation of CWPF programs.

The absence of standardized PROMs in irradiated cohorts remains an important limitation of the current literature, restricting assessment of long-term aesthetic and QoL outcomes. This issue is being addressed by the ongoing PARTBRECON-Pro study [50,51], an international multicenter prospective cohort trial incorporating radiation oncology input and PROMs collected preoperatively and six months after RT.

##### Imaging Surveillance

Another underexplored aspect of CWPF reconstruction relates to postoperative imaging surveillance. Although breast preservation facilitates routine mammographic follow-up, flap-related changes such as fat necrosis, fibrosis, oil cyst formation and postoperative architectural distortion may occasionally complicate radiological interpretation. To date, no dedicated studies have evaluated imaging findings following CWPF reconstruction or their impact on postoperative surveillance accuracy. Improved collaboration between breast surgeons and breast radiologists, together with dedicated imaging-based studies, may help establish standardized surveillance protocols and reduce diagnostic uncertainty.

#### 4.5.4. Variability in Margin Assessment and Surgical Workflows

Margin control represents a critical aspect of CWPF reconstruction, as re-excision may negatively affect both oncological workflow and aesthetic outcomes. In the present institutional series, all patients underwent routine intraoperative frozen section analysis, and no patient required re-excision following final histopathological examination. This finding contrasts favorably with published literature, where re-excision rates from approximately 9% to 16% have been reported across systematic reviews and multicenter cohorts [2,4,5,6,12,18].

However, the role of frozen section assessment remains controversial [52]. While some centers incorporate routine intraoperative margin evaluation [8,17,25,32,36,53], others rely solely on final histopathology due to concerns regarding increased operative time, resource utilization and potential discordance between frozen and permanent sections. Indeed, previous studies, such as Munhoz et al., have demonstrated cases of positive final margins, in 5.5% of patients despite negative intraoperative assessment, highlighting the imperfect sensitivity of frozen section analysis [53].

This lack of standardization complicates interpretation of the available evidence. Differences in intraoperative margin assessment strategies may partly account for the variability in reported re-excision rates across studies, making direct comparisons challenging. Development of consensus-based recommendations regarding the role of frozen section assessment in CWPF reconstruction would improve both clinical practice and the comparability of future studies.

Nevertheless, our experience suggests that systematic intraoperative margin assessment may contribute to the exceptionally low re-excision rate observed in this cohort and may facilitate the safe application of CWPF reconstruction, particularly in patients requiring larger resections or presenting with challenging tumor locations.

#### 4.5.5. Educational and Institutional Barriers to Adoption

Despite the growing body of evidence supporting the safety, versatility and aesthetic advantages of CWPF reconstruction, widespread adoption remains limited [1,3,6,8,12,18,27,30,33,36]. The findings of the present review reveal a notable discrepancy between the academic validation of the technique and its implementation in routine clinical practice. While most published studies report promising surgical, oncological and patient-reported outcomes [1,3,6,8,12,18,27,30,33,36], relatively few have explored the practical barriers that prevent broader dissemination [4,34].

The international PERDITA survey conducted by Karakatsanis et al. provides valuable insight into this evidence–adoption gap [4]. Although 88.4% of respondents considered CWPF reconstruction a desirable component of contemporary OBS, only a minority reported actively performing these procedures. The authors concluded there is a discrepancy between perceived importance and availability, which is indicative of ongoing underutilization. Interestingly, no distinct “profile” of skills, background or experience reliably identified surgeons who routinely undertake CWPF reconstruction. While surgeons performing CWPFs were more likely to be senior, formally trained in oncoplastic surgery and working in larger units with reconstructive services, cluster and latent class analyses failed to demonstrate a clear pattern predicting adoption. These findings suggest that implementation depends on a complex interaction of training exposure, institutional support, clinical experience and local practice culture rather than any single determinant.

The survey further demonstrated that the strongest predictor of CWPF availability within a unit was the presence of a free-flap breast reconstruction service, highlighting the importance of reconstructive infrastructure and specialist expertise [4]. Nevertheless, significant discordance remained between the availability of free-flap reconstruction and CWPF services, suggesting that technical familiarity and local experience may be equally important determinants of adoption [4].

A major barrier identified by the survey relates to training and skill acquisition. Two-thirds of respondents reported no exposure to CWPF surgery during formal training, despite formal oncoplastic training being the only factor independently associated with exposure to the technique [4]. Furthermore, more than one-third felt that existing training pathways would not necessarily provide sufficient experience to perform CWPF reconstruction independently [4]. Importantly, over 60% of respondents expressed a need for further training in key procedural steps, including patient selection, flap design, perforator localization, perforator dissection and flap mobilization [4]. Notably, these educational needs persisted even among surgeons already performing CWPF procedures, reflecting the perceived technical complexity of the technique.

The learning curve associated with CWPF reconstruction likely represents an additional obstacle to wider dissemination. Successful implementation requires familiarity with perforator anatomy, preoperative planning, flap harvest, perforator preservation and intraoperative decision-making. Unlike more established oncoplastic techniques such as therapeutic mammoplasty, CWPF reconstruction lacks universally accepted competency frameworks, assessment tools or structured training pathways. Consequently, surgeons may be reluctant to adopt the technique despite recognizing its clinical value.

While the learning curve associated with CWPF reconstruction is frequently cited as a barrier to wider implementation, evidence evaluating how this challenge can be overcome remains scarce. In a low-resource setting, Yadav et al. [34] evaluated institutional adoption of local perforator flap techniques and demonstrated that integration into resident training programs and clinical care pathways was associated with progressive increases in utilization over time, underscoring the role of structured educational and organizational frameworks in facilitating implementation. To our knowledge, this represents the only report to specifically examining institutional adoption processes of CWPF. Rather than focusing solely on clinical outcomes, the authors applied a composite framework encompassing clinical utilization, surgical outcomes, surgeon uptake and policy-level implementation. Over a three-year period, use of local perforator flaps increased from 12% to 33% of BCS, alongside formal incorporation of the technique into preoperative counselling and surgical training pathways. A measurable learning curve was also observed, with progressive reductions in operative time and complication rates across sequential cases. Importantly, implementation was achieved without reliance on advanced technologies such as Doppler localization or fluorescence imaging, supporting the feasibility of CWPF adoption even in resource-constrained environments when appropriate training and institutional support are in place. Although conducted in a single center, these findings suggest that successful dissemination of CWPF techniques may depend not only on surgical expertise but also on deliberate integration into educational structures and clinical workflows [33].

Uncertainty regarding patient selection may further contribute to underutilization. As discussed previously, indications for CWPF reconstruction remain incompletely standardized, and decision-making frequently relies on surgeon judgement rather than validated selection criteria. This variability may reduce confidence among surgeons with limited experience and create additional barriers to implementation.

Institutional factors also appear to play an important role. CWPF reconstruction remains concentrated within specialized centers with dedicated oncoplastic expertise, limiting exposure during residency and fellowship training. Variability in multidisciplinary team familiarity, operative logistics, RT planning pathways and local reconstructive culture may further influence adoption. In many settings, surgeons may continue to favor more familiar alternatives such as therapeutic mammoplasty, IBR, LD flap reconstruction or mastectomy, particularly when access to mentorship and specialized training is limited.

Finally, the substantial heterogeneity observed throughout the CWPF literature may itself impede dissemination. Variability in indications, flap nomenclature, operative techniques, complication reporting, aesthetic assessment methods and follow-up protocols complicates interpretation of the available evidence and limits the development of consensus recommendations. As a result, although CWPF reconstruction has accumulated considerable academic support, translation into routine clinical practice has lagged.

Taken together, these findings suggest that the underutilization of CWPF reconstruction is unlikely to reflect concerns regarding safety, efficacy or oncological outcomes. Rather, it appears to arise from a combination of limited training opportunities, absence of standardized educational pathways, uncertainty regarding patient selection, institutional barriers and lack of consensus regarding implementation. CWPF reconstruction may therefore be viewed as a high-value but relatively low-access oncoplastic technique. Addressing these barriers through structured training programs, competency-based curricula, consensus guidelines and wider dissemination of expertise may represent the next critical step in facilitating broader adoption.

### 4.6. Geographical Variability and Diffusion of CWPF Reconstruction

Beyond these intrinsic barriers, CWPF uptake also appears to vary considerably across healthcare systems and geographical regions. There is currently no dedicated registry systematically capturing the uptake of CWPF or other oncoplastic techniques at a national level. As a result, available evidence is largely derived from observational studies, survey data and expert opinion. Nonetheless, the existing literature suggests that CWPF adoption is most consistently reported in selected tertiary breast units, particularly within parts of Europe, including the United Kingdom, Sweden, Belgium and the Netherlands. These settings are characterized by established oncoplastic fellowship programs, early contribution to CWPF development in the literature and closer integration between breast oncological and reconstructive surgical services.

The **United Kingdom** represents one of the most developed models of CWPF integration, where oncoplastic principles have been incorporated early into national training pathways, including structured programs such as the Association of Breast Surgery initiatives and national oncoplastic training curriculum. This high level of oncoplastic penetration is reflected in earlier UK survey data, which reported that approximately 39% of breast surgeons offered partial mastectomy with reconstruction, defined as an excision of more than 20% of breast tissue where mastectomy represents the alternative [54]. The progressive expansion of breast-conserving strategies in the UK is further illustrated by the evolution of “extreme oncoplastic surgery,” which has extended indications for breast conservation to include larger, multifocal or multicentric tumors in selected patients who might otherwise have been considered for mastectomy [19,55]. This reflects a broader cultural and technical shift towards more extensive breast conservation facilitated by oncoplastic techniques.

By contrast, within **Europe**, lower levels of reported adoption appear to be present in several Central, Eastern and Southeastern European countries, including Greece, Portugal, Romania, Bulgaria, Serbia and Croatia. In lower-adoption settings, breast cancer surgery may still rely more frequently on mastectomy with or without implant-based reconstruction, as well as established oncoplastic volume displacement techniques [56]. However, these observations are primarily derived from indirect indicators such as variability in fellowship infrastructure, oncoplastic training exposure and rates of IBR, rather than formal national registries. The 2023 PERDITA survey data similarly highlight substantial regional variation within Europe, with 103 surgeons reporting CWPF use versus 170 non-users among European respondents, although country-level comparisons were not reported [4]. Multivariable analysis suggested that factors such as formal training exposure, unit size and service availability were more influential than geographical region alone [4]. The current European landscape is summarized in Table 7, highlighting heterogeneous CWPF uptake across regions.

In **South America**, CWPF adoption appears to be increasing, although it remains concentrated in major oncoplastic and breast reconstruction centers in countries such as Argentina, Brazil, Colombia and Chile. Historically, breast reconstruction in the region has relied heavily on implant-based techniques and traditional autologous flaps, including LD and transverse rectus abdominis myocutaneous (TRAM) and deep inferior epigastric perforator (DIEP) flaps. More recently, however, there has been a gradual shift towards perforator-based PBR, driven by both the expansion of BCS and limitations in microsurgical resources within public healthcare systems. Argentina has emerged as an important contributor to CWPF development and dissemination within the region, supported by early technical innovations, influential surgical pioneers and specialized centers where pedicled perforator techniques have become an established components of reconstructive practice and training [46]. Brazil similarly represents one of the most developed oncoplastic environments in South America, characterized by a dual healthcare structure. While advanced autologous reconstruction is widely available in high-volume private centers, access remains more variable within the public healthcare sector, resulting in uneven adoption of CWPF techniques across different settings. Despite this growth, wider dissemination remains limited. Barriers to broader adoption include the lack of formalized training pathways, variability in surgical infrastructure across public and private sectors and persistent reliance on mastectomy-based strategies or established volume displacement techniques in many institutions [4].

In **North America**, CWPF use is expanding, particularly within specialized oncological and reconstructive centers. However, broader dissemination appears to be influenced by structural factors within surgical practice. Breast surgeons typically perform oncological resection, while plastic surgeons are primarily responsible for reconstruction and this division of roles may limit cross-disciplinary collaboration and slow adoption in smaller community-based hospitals. In addition, reimbursement frameworks in some healthcare systems may influence the relative uptake of autologous reconstructive options compared with alternative reconstructive strategies [57,58].

In parts of **Asia**, **Africa** and **other regions**, emerging evidence suggests increasing interest and gradual adoption of CWPF techniques, particularly within high-volume oncology centers. However, access to formal training, resource availability and institutional experience remain key limiting factors, resulting in variable implementation across and within countries. Among these regions, India appears to demonstrate a comparatively more established and growing experience with CWPF techniques [33,34,36], while countries such as China [17], South Korea [8], Egypt [32] and Pakistan [30,31] report emerging but more heterogeneous adoption, primarily concentrated within major tertiary oncology centers.

A consistent observation across all regions is that CWPF adoption is often center-dependent rather than country-dependent. Even in regions with overall limited penetration, selected tertiary academic or private institutions may demonstrate high levels of expertise and routine implementation, whereas the technique may be rarely performed in surrounding hospitals. This pattern underscores the importance of local expertise, training exposure and institutional infrastructure as key drivers of diffusion, rather than purely national-level determinants.

### 4.7. Limitations

Several limitations should be acknowledged when interpreting the findings of the present study. These limitations relate both to the inherent constraints of our institutional cohort and to the methodological heterogeneity of the available literature on CWPF reconstruction.

First, the institutional component of this study represents a retrospective, single-center experience, performed within a single institution by a single trained surgeon. Although our cohort constitutes one of the largest single-center CWPF series reported to date in Greece, this design may introduce both selection and institutional bias. Specifically, patients were selected based on clinical suitability for CWPF reconstruction, which may limit the applicability of the findings to the broader population of breast cancer patients undergoing BCS. In addition, outcomes may reflect local surgical expertise, case-mix and institutional protocols, thereby limiting generalizability to other settings. Furthermore, no comparator group was available, preventing direct comparison with alternative oncoplastic approaches, such as therapeutic mammoplasty, LD flap reconstruction or mastectomy-based strategies.

Another important limitation relates to the available clinical data. Formal PROMs, such as the Breast-Q, were not routinely collected, precluding objective assessment of patient satisfaction and QoL outcomes. Similarly, several potentially relevant clinicopathological variables were not consistently recorded, including smoking status, diabetes mellitus and other comorbidities that may influence surgical outcomes. Data regarding breast ptosis, molecular tumor subtype, TNM staging and other patient-specific factors were also incomplete, limiting the ability to perform more detailed subgroup analyses and adjustment for potential confounding variables.

Although oncological outcomes were encouraging, follow-up duration was not uniformly available across the cohort, limiting assessment of long-term local control, recurrence patterns and survival outcomes. Importantly, while margin positivity rates were zero, long-term oncological endpoints were not systematically evaluated and therefore require longer follow-up for definitive assessment. In addition, detailed RT-related data, including boost volumes, treatment planning parameters and radiation-associated toxicity, were not systematically collected. Consequently, the interaction between CWPF reconstruction and adjuvant RT could not be adequately evaluated within the present cohort.

The literature synthesis component of this study is also subject to several limitations. As this was a structured narrative review rather than a systematic review or meta-analysis, the selection and qualitative interpretation of the literature may retain a degree of reviewer-dependent subjectivity, despite independent assessment by two reviewers and resolution of disagreements by consensus. In addition, no formal quantitative synthesis was undertaken. The substantial heterogeneity of the available evidence with respect to study design, patient populations, flap selection, operative techniques, outcome definitions and follow-up duration limits direct comparison across studies and precludes robust pooled estimates. The available evidence remains dominated by retrospective cohort studies, with relatively few prospective investigations and an absence of randomized comparative trials (RCTs), thereby restricting the overall level of evidence.

Moreover, only a small number of systematic reviews and pooled analysis are available, reflecting the relatively early stage of evidence consolidation in CWPF reconstruction. Considerable heterogeneity exists across published studies regarding patient selection criteria, flap selection, operative techniques, definitions of complications, margin assessment strategies, aesthetic evaluation methods and duration of follow-up. Such variability limits direct comparison between studies and precludes robust quantitative synthesis.

In addition, outcome reporting remains inconsistent throughout the CWPF literature. Definitions of complications such as seroma, fat necrosis, wound morbidity and flap-related events vary substantially between studies, while the use of validated PROMs remains limited. Similarly, functional outcomes, including patient-reported symptoms such as lateral chest wall tightness or discomfort during arm elevation, have not been consistently assessed or reported across published CWPF series, further limiting meaningful comparisons between cohorts. Follow-up durations are frequently short and inconsistent, reducing confidence in long-term oncological and aesthetic conclusions. Publication bias may also be present, as most published CWPF series originate from high-volume specialist centers with dedicated oncoplastic expertise, potentially limiting the representation of real-world outcomes from lower-volume units. Overall, these limitations reflect the lack of standardized reporting frameworks and consensus definitions within the current CWPF literature, which should be considered when interpreting the available evidence.

### 4.8. Strengths of the Study

The present study has several strengths. To our knowledge, it represents one of the largest single-center CWPF series reported in the literature, contributing valuable real-world data from a high-volume institutional setting. The combined approach of a narrative literature synthesis and an institutional cohort allow for both contextualization of published evidence and validation through real-world experience. Unlike most prior reviews that focus predominantly on surgical and oncological outcomes, this work incorporates an implementation perspective, including evidence from the PERDITA/Karakatsanis survey [4], directly addressing the evidence–practice gap and the issue of underutilization. In addition, the inclusion of studies from diverse geographical and clinical settings enhances the external validity of the findings and provides a broad overview of contemporary CWPF practice. At an institutional level, all procedures were performed within a standardized oncoplastic workflow by a dedicated surgical team, ensuring procedural consistency and reproducibility.

### 4.9. Future Research

Future research should primarily aim to address the existing evidence–adoption gap in CWPF reconstruction by strengthening both the methodological quality of the literature and the practical frameworks supporting clinical implementation. A key priority is the development of standardized training pathways in OBS, ideally through structured curricula and competency-based programs.

In parallel, further prospective multicenter registries and, where feasible, comparative studies or RCTs are needed to strengthen the evidence base, particularly regarding long-term oncological outcomes through extended follow-up, survival endpoints and durability of reconstructive results.

Equally important is the incorporation of validated PROMs and standardized aesthetic assessment tools, such as Breast-Q-based or equivalent instruments. This would allow more robust evaluation of patient satisfaction and QoL, facilitating meaningful comparisons across techniques and institutions.

Radiological follow-up after CWPF reconstruction also warrants dedicated investigation. Standardized imaging protocols are needed to better characterize postoperative changes such as fat necrosis, fibrosis and architectural distortion, and to optimize communication between breast surgeons and radiologists in surveillance settings.

Furthermore, there is a need for clearer and more consistent patient selection algorithms, incorporating tumor characteristics, breast morphology and volumetric assessment. The development of decision-support tools may improve reproducibility and assist in identifying patients most likely to benefit from CWPF reconstruction. An additional area warranting investigation is the potential role of CWPF reconstruction in expanding eligibility for BCS and reducing the need for mastectomy in patients with challenging tumor-to-breast volume ratios or unfavorable tumor locations.

Importantly, future studies should also focus on implementation research, exploring barriers and facilitators to adoption in real-world clinical settings. This includes cost-effectiveness analyses, institutional readiness and the role of multidisciplinary team structures in supporting CWPF delivery. Such work is essential to understand not only whether CWPF reconstruction is effective but why its uptake remains limited despite optimal outcomes.

## 5. Conclusions

CWPF reconstruction represents a safe, versatile and oncologically safe option for PBR. Despite their advantages, CWPFs appear to remain underutilized in routine practice, as suggested by the limited literature addressing their dissemination and implementation [4,34]. Variability in patient selection, lack of standardization, limited training exposure and heterogeneity within the available evidence base may contribute to this adoption gap. Bridging this evidence–practice gap through education, standardization and collaborative research through prospective multi-institutional validation studies may represent the most important step towards broader adoption of CWPF techniques and optimize patient access to these techniques.

## Figures and Tables

**Figure 1:**
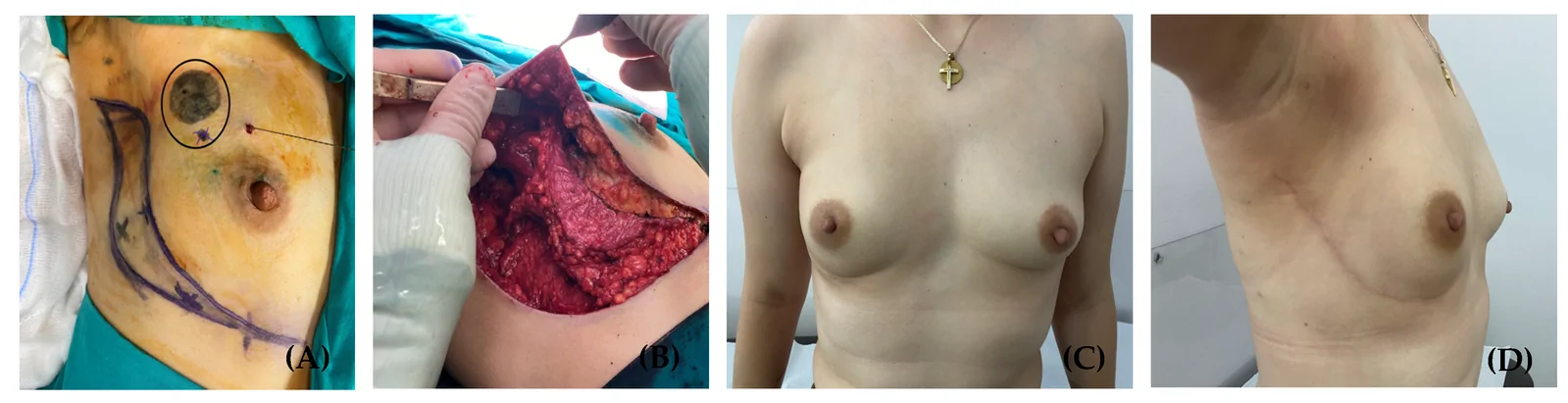
A 44-year-old with small breasts. Right lateral intercostal artery perforator (LICAP). (**A**) Preoperative. The black circle delineates the “en bloc” excision. Two distinct tumors are included: the main tumor 3 cm and an adjacent satellite nodule. This comprehensive excision represents the total volume of breast tissue removed to achieve clear margins. (**B**) Intraoperative (**C**,**D**) 12 months after radiotherapy.

**Figure 2:**
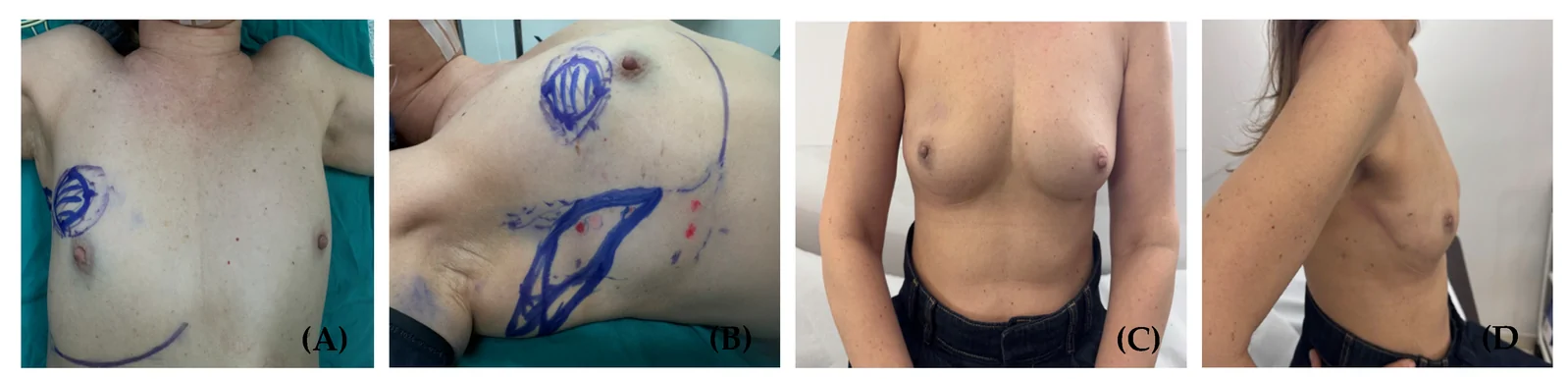
A 58-year-old with small, non-ptotic breasts. Right lateral thoracic artery perforator (LTAP). (**A**,**B**) Preoperative (**C**,**D**) 12 months after radiotherapy.

**Figure 3:**
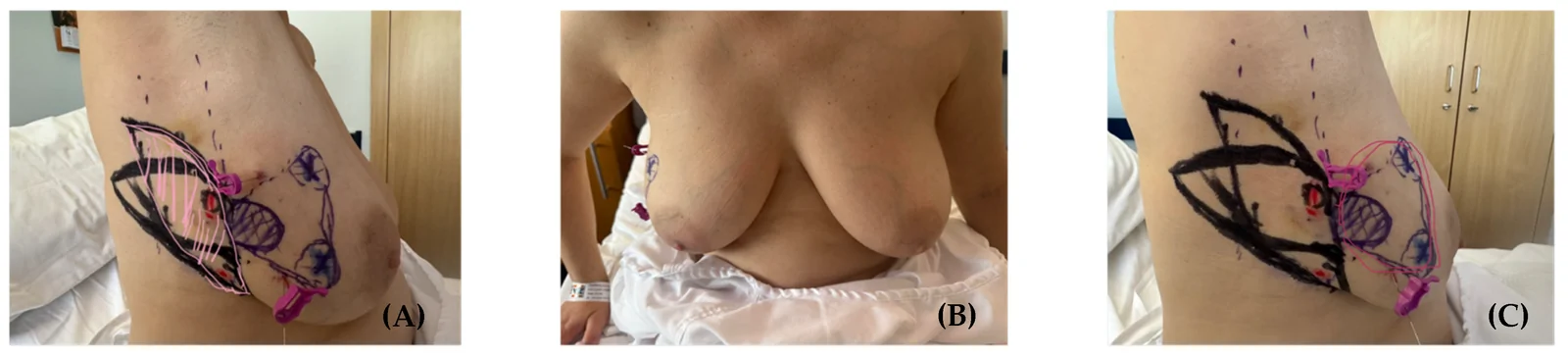
Identification of the perforators and flaps design. Preoperative markings for breast-conserving surgery and oncoplastic reconstruction. (**A**) Lateral view demonstrating the planned local flap. The pink dots indicate the location of the perforator artery and the shaded area indicates the flap selected for reconstruction of the anticipated defect. (**B**) Frontal preoperative clinical view of the breasts. (**C**) Lateral view demonstrating the planned en bloc excision. The pink circle delineates the area to be removed en bloc, encompassing a triple-focus malignancy: an invasive carcinoma at the lateral margin and two foci of ductal carcinoma in situ (DCIS), localized by hook-wire guidance.

**Figure 4:**
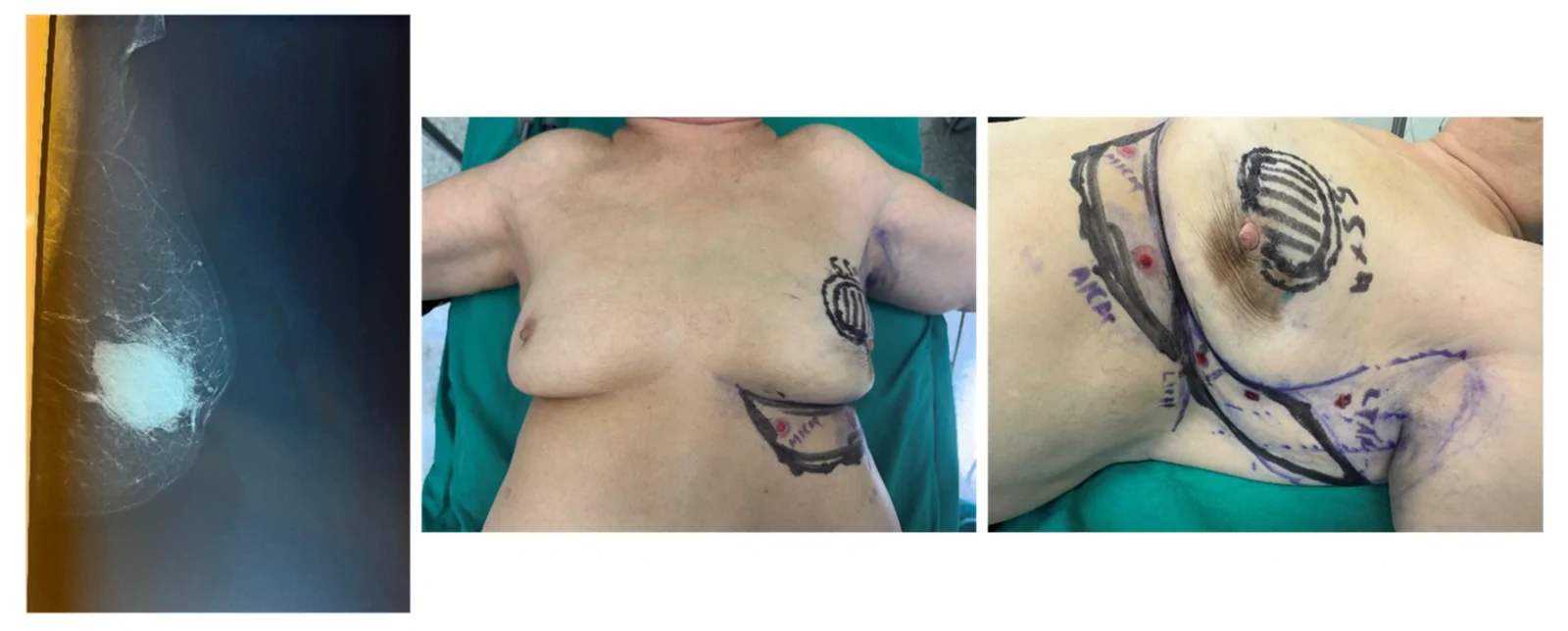
Multiple chest wall perforators are identified and mapped preoperatively due to the large tumor size (5.5 cm). The available perforator options with a medial-to-lateral distribution include medial intercostal artery perforator (MICAP), anterior intercostal artery perforator (AICAP), lateral intercostal artery perforator (LICAP) and lateral thoracic artery perforator (LTAP), allowing tailored selection of the most appropriate reconstructive flap based on defect location, perfusion requirements and breast volume replacement needs.

**Figure 5:**
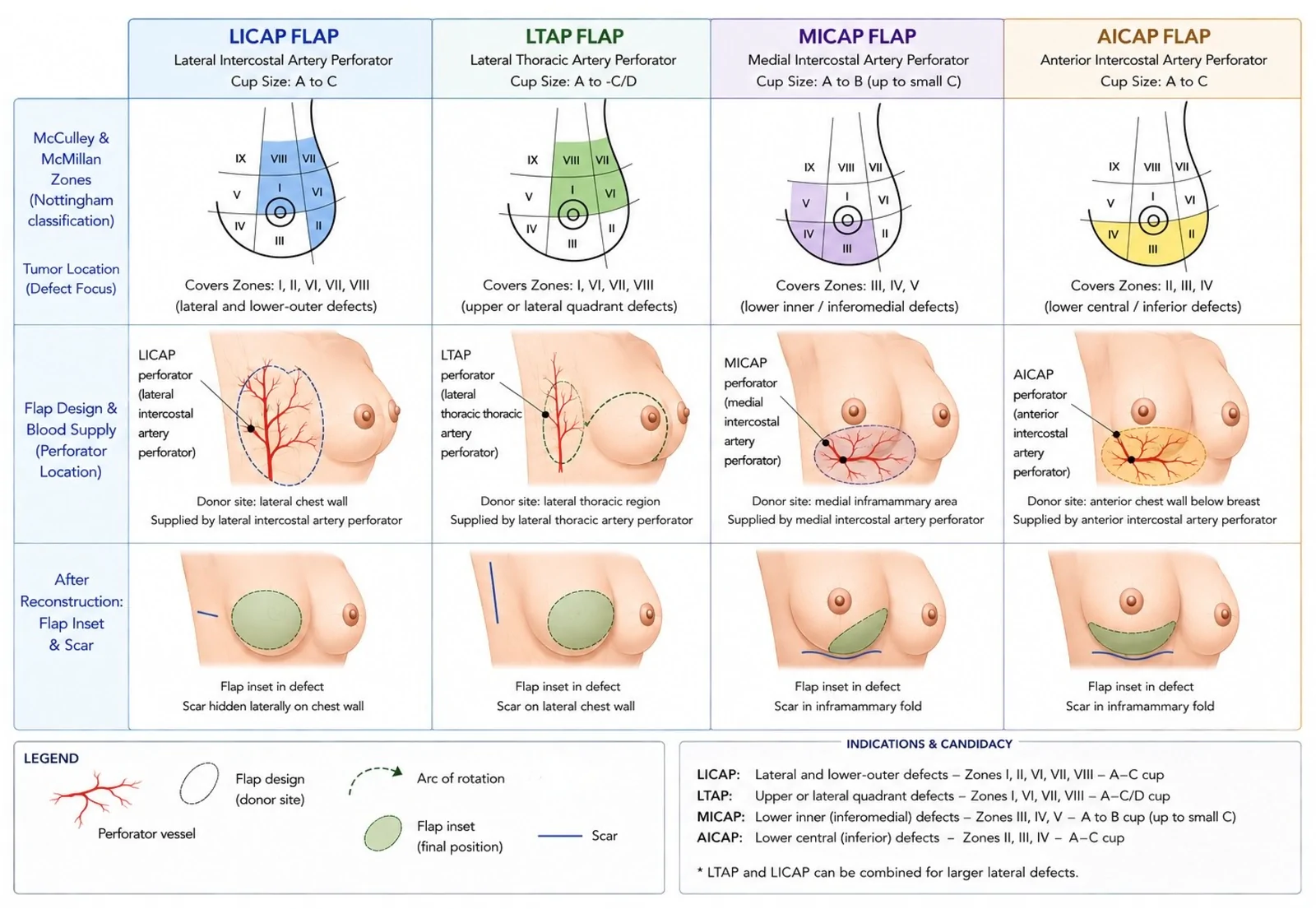
Chest wall perforator flap selection is primarily based on tumor location and defect characteristics, in accordance with the McCulley–McMillan zones and the corresponding breast cup size, as illustrated in the figure. LICAP flaps are used for lateral breast defects. The tissue is harvested from the lateral chest wall and transferred into the breast to replace the excised volume, with the resulting scar typically hidden on the lateral thoracic wall behind the breast. LTAP flaps are indicated for upper or lateral quadrant defects. The donor tissue is taken from the lateral thoracic region, and the scar is also located laterally, with the flap used to restore volume within the breast. AICAP flaps are used for lower or lower central breast defects. Tissue is harvested from the inframammary fold, used as a turnover flap, with the scar concealed within the natural breast crease. In cases requiring axillary lymph node surgery, a small additional axillary incision may be present. MICAP flaps are primarily used for medial or lower medial quadrant defects. Similar to AICAP flaps, tissue is taken from the inframammary region but is transposed more medially to restore volume in the medial breast. In selected cases with larger defects, combinations of perforator flaps may be used to achieve adequate volume replacement and reliable perfusion. LTAP and LICAP flaps are frequently combined for extended lateral defects. Particular attention is given to LTAP availability, as it may be absent in approximately 10% of cases due to anatomical variability or vascular limitations. In addition, potential compromise of LTAP perfusion following axillary dissection is considered when selecting between LICAP and LTAP flaps.

**Figure 6:**
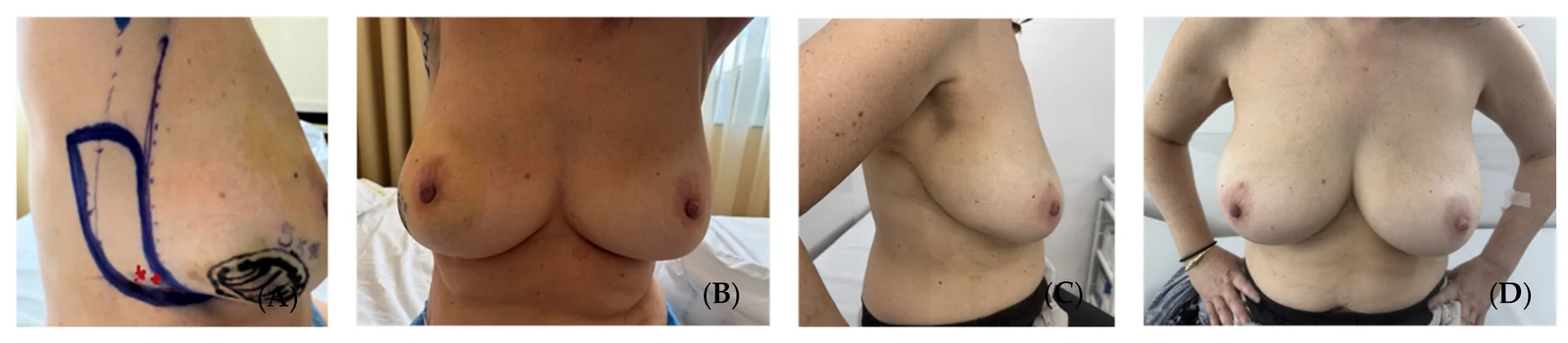
A 53-year-old with a right lateral intercostal artery perforator (LICAP). (**A**,**B**) Preoperative (**C**,**D**) 12 months after radiotherapy.

**Figure 7:**
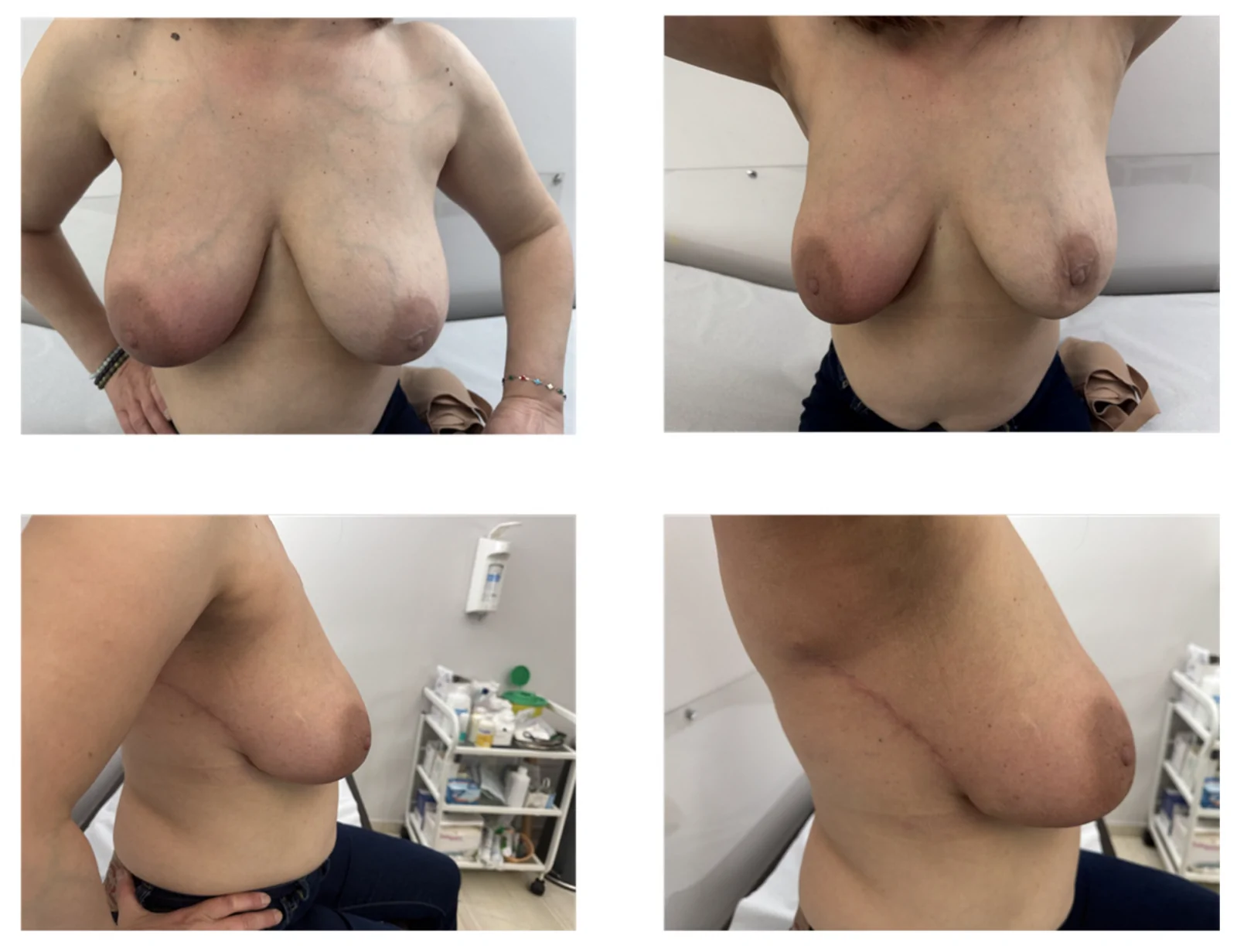
A 47-year-old patient with a right lateral thoracic artery perforator (LTAP) 12 months after radiotherapy.

**Figure 8:**
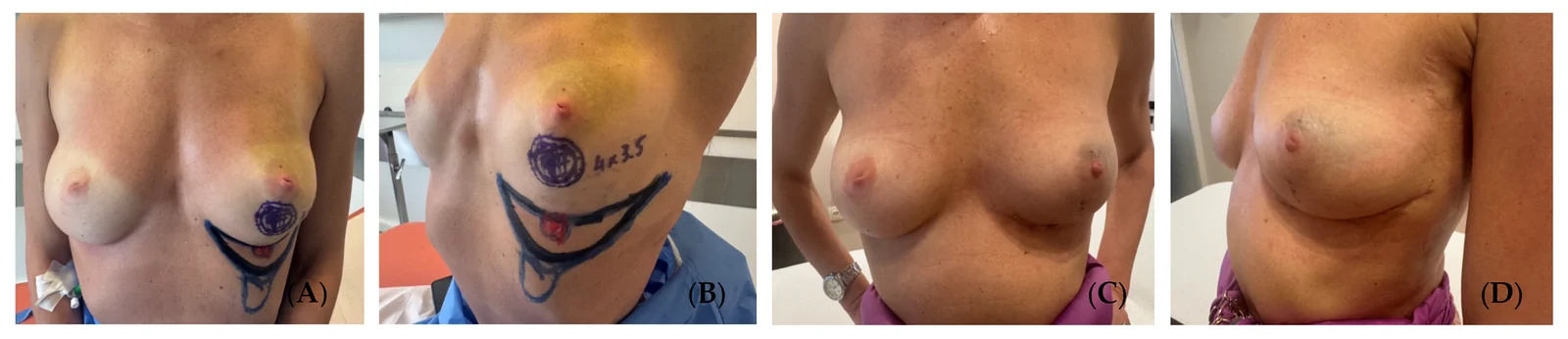
A 38-year-old with a left anterior intercostal artery perforator (AICAP). (**A**,**B**) Preoperative (**C**,**D**) 6 months after radiotherapy.

**Figure 9:**
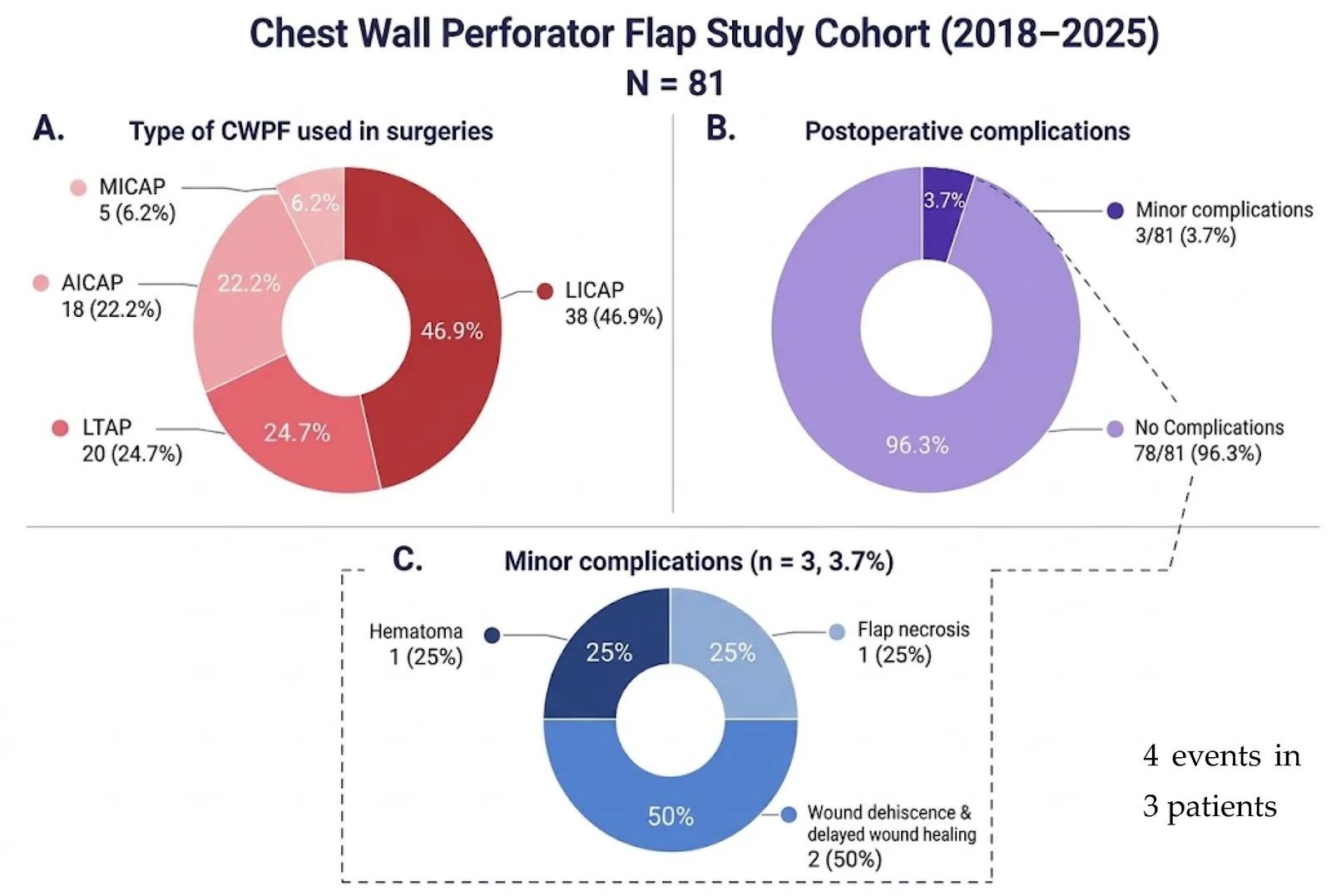
Chest wall perforator flap (CWPF) study cohort (2018–2025), *n* = 81. (**A**) Distribution of flap types used in breast reconstruction, demonstrating lateral intercostal artery perforator (LICAP) as the most frequently utilized flap (46.9%), followed by lateral thoracic artery perforator (LTAP) (24.7%), anterior intercostal artery perforator (AICAP) (22.2%) and medial intercostal artery perforator (MICAP) (6.2%). (**B**) Overall postoperative outcomes, showing a low complication rate with 96.3% of patients experiencing no complications and 3.7% presenting minor complications. (**C**) Breakdown of minor complications (zoomed-in view of Panel (**B**)), including hematoma (1.2%), wound dehiscence with delayed wound healing (2.5%) and flap necrosis (1.2%).

**Table 1:** Characteristics of included studies.

First Author	Year	Study Period	Country	Study Design	Centers	Sample Size (*N*)	Breast Cup Size	Most Used Flap	Follow-Up	Complication Rate	Re-Excision (CtM)	LRR	Distant Recurrence	Oncological Outcomes	Key Notes
Orabi et al. [32]	2022	2019–2020	Egypt	Prospective	Single	26	B (mostly)–D	LICAP	Short	7.7%	0	NR	NR		3-Month Mastectomy Questionnaire
Agrawal et al. [33]	2024	2015–2022	India	Retrospective	Single	150	A 10 (6.7%) B 74 (49.2%) C 52 (34.7%) D 13 (8.7%) F 1 (0.7%)	LICAP	Median 16 months (IQR 9–37)	Acceptable	6% (1 CtM)	2	4	DFS 86.4% OS 94.7%	Ptosis recorded
Xie et al. [17]	2025	2021–2022	China	Retrospective	Single	22	NR	LTAP	Median 14.5 months	1/22	0	1	NR		- Increased hospital stay 7–15 days - Kyungpook National University Hospital Breast Satisfaction Survey
Pinto et al. [29]	2023	2020–2022	Portugal	Prospective	Single	45	A 7 (15.6%) B 25 (55.6%) C 12 (26.7%) D 1 (2.2%)	LICAP	12 months	37.8%	8.9%	NR	NR		Comorbidities & Ptosis Grade reported
van Zeelst et al. [35]	2024	2019–2023	Netherlands	Retrospective	Single	42	A 13 (31%) B 14 (33.3%) C 6 (14.3%) D 2 (4.8%)	LICAP	>1 year	16.7%	0	NR	NR		- Breast-Q -95.2% - RT—low incidenced of fat necrosis and breast edema confirm that intended RT should not preclude the use of CWPF
Agrawal et al. [1]	2020	2017–2019	India	Prospective	Single	40	B 30% C 52.5%	LICAP	Median 18 months (IQR 10–22)	12.8%	10% (1 CtM)	0 (1 regional/nodal)	1		ICG use after a flap loss satisfaction assessment
Soumian et al. [12]	2020	2015–2018	UK	Prospective	Multi	112	NR	LICAP ± LTAP	Median 15 months	7.14%	13.39% (1 CtM)	1	1		
Rankin et al. [22]	2024	2017–2023	UK	Retrospective	Single	30	A–D mostly B & C	LICAP	Median 16 months (1–64)	40%	0	NR	NR		
Koppiker et al. [36]	2025	2018–2023	India	Retrospective	Single	200 pts (203 flaps)	NR	LICAP	Median 27 months	11%	0	6.6%	5.4%	OS 97.55% DFS 92.15% Overall Mortality 3.5%	- 59 patients post-RT reactions reported - Breast-Q
Yadav et al. [34]	2025	2022–2025	India	Prospective	Single	28	NR	LICAP	NR	10.7%	0	Not studied	NR		- Composite Adoption Score - Learning curve - Adoption
Agrawal et al. [6]	2023	2011–2021	UK	Retrospective	Multi (15)	505	A–C (73.4%) B mostly	LICAP	Median 23 months (IQR 11–39)	12% seroma mostly	15.9%	1% (0.6% regional/nodal)	3.2%		- 1.2% symmetrization surgery - 2.6% lipomodelling and/or scar revision
Hamdi et al. [28]	2006	2001–2004	Belgium	Prospective	Single	20 flaps (16 pts) 18 CWPF	NR	LICAP	NR	NR	NR	NR	NR		
Kim et al. [8]	2018	2011–2014	Korea	Retrospective	Single	33	NR	LICAP 19 TDAP 14	Mean 25.2 months (±8.69)	15.15%	9.1%	0	NR		Kyungpook National University Hospital Breast Satisfaction Survey
Roy et al. [27]	2017	2011–2015	UK	Prospective	Single	20	A 1 B 7 C 5 D 7	LICAP LTAP	Median 23 months (17–38)	Low	Clear margins assurance due to 2-stage approach	0	2		- 2-stage approach - Reconstruction 2–4 weeks after initial surgery
Roy et al. [20]	2021	2011–2018	UK	Retrospective	Single	105	AA 3 A 7 B 33 C 39 D 23	LICAP	Median 4.5 years (2.5–8.5 years)	19%	10% (4% CtM)	2%	10.5%	DFS 86% DDFS 89% OS 94.8%	Breast-Q
Kim et al. [25]	2019	2011–2016	Korea	Retrospective	Single	40	NR	LICAP (6th + 7th) only	Mean 25.6 months (±8.69)	22.5%	9.1%	NR	NR		
Dube et al. [23]	2023	2018–2022	UK	Retrospective	Single	81	A 6 (7.4%) B 23 (28.3%) C 31 (38.2%) D 11 (13.6%) DD 9 (11.1%) FF 1 (1.2%)	LICAP	3 weeks	3.7% Flap complication 4.9% Flap Shrinkage 3.7% Hematoma 17.3% Other	12.3% (2.5% CtM)	NR	NR		
McCulley et al. [15]	2015	2007–2013	UK	Prospective	Single	75	NR	LICAP	NR	NR	NR	NR	NR		
Munhoz et al. [24]	2011	1999–2009	Brazil	Prospective	Single	13	NR	LICAP	Mean 32 months (18–45)	23%	0	0	NR		
Hamdi et al. [13]	2004	2000–2003	Belgium	Retrospective	Single	21 CWPF (*n* = 31—10 LD)	NR	TDAP	NR	9.5%	NR	NR	NR		
Schaverian et al. [37]	2020	2016–2017	US—Texas	Retrospective	Single	21	B 7 (33.3%) C 11 (52.4%) D 3 (14.3%)	LICAP	Mean 17.5 months ± 5.7	0	14.3%	NR	NR		1 need for symmmetrization (4.7%)
Karakatsanis et al. [3]	2024		International (Sweden, UK, Australia)	Prospective	Multi	603	NR	NR	Median 22 months (3–98) (IQR 16–39)	8.6%	15.9% (1.5% CtM)	1.9%	4.9%	BC mortality 4.9%	- 3.6% revision surgery (dog ear, lipofillling etc) - Suggested nomogram for patient selection
Zeeshan et al. [30]	2022	2018–2021	Pakistan	Retrospective	Single	25	NR	LICAP 8 LTAP 10 AICAP7	NR	8%	1 CtM	0	1		Breast-Q
Laroiya et al. [10]	2023	2017–2022	Birmingham	Retrospective	Single	145 (11 novel)	NR	LICAP	Median 8 months (2–17)	18.19% (2/11)	NR	0	0		- 10 uses of PROMs - Even WBR with CWPF
Khan and Mathew [11]	2021	2016–2021	UK	Prospective	Single	30	80% A, B, C 20% D & above	LICAP + LTAP (combo)	Median 22 months (1–49)	3% needing intervention	17% (0 CtM)	0	10%		
Kabeer et al. [26]	2022	2016–2021	UK	Retrospective	Single	152	90% A-C	LICAP	Median 23 months (4–61)	10.46%	11.11% (3 CtM)	0.65%	4.6%		
Hu et al. [21]	2019	2013–2014	UK	Retrospective	Single	18	NR	NR	Median 4 years Mean 3.83 years (3–5)	NR	1	NR	NR		- Control group WLE - Same postoperative qualitative findings on mammograms
Soomro et al. [31]	2025	2020–2024	Pakistan	Retrospective	Single	33	NR	LICAP only	Short	9%	NR	NR	NR		SDC1 PROM tool
Meybodi et al. [16]	2019	2015–2016	Australia	Prospective	Single	22	NR	Modified LICAP	2 months (1–12)	18.18%	18%				
Legarda et al. [9]	2025	2016–2023	Israel	Retrospective	Single	23	B or C cup in all cases	TDAP	Mean 19 months (±20)	21.7%	1	NR	NR		
Reid et al. [28]	2025	2016–2024	UK	Retrospective	Single	290	*N* = 115 AA 1 (0.9%) A 10 (8.7%) B 24 (20.9%) C 24 (20.9%) D 24 (20.9%) E 13 (11.3%) F 13 (11.3%) H 3 (2.6%)	LICAP	Median 3.1 years (IQR 1.4–3.9)	28.6% (*n* = 276)	15.6% (1.5% CtM)	2.9% (4.2% in subset analysis *n* = 96 follow-up 5.1 y)	3.62%	DFS 93.8% DDFS 95.3% Overall Mortality 2.9% BC mortality 2.2%	

CtM, completion to mastectomy; LRR, local recurrence rate; LICAP, lateral intercostal artery perforator; NR, not reported; IQR, interquartile range; DFS, disease free survival; OS, overall survival; LTAP, lateral thoracic artery perforator; RT, radiotherapy; CWPF, chest-wall perforator flap; ICG, indocyanine green; PTS, patients; PROMs, patient-reported outcome measures; TDAP, thoracodorsal artery perforator; DDFS, distant disease free survival; BC, Breast cancer; AICAP, anterior intercostal artery perforator; WBR, whole breast reconstruction; WLE, wide local excision; SDC1, structure data capture 1.

**Table 2:** Characteristics of recent Systematic Reviews.

First Author	Year	Meta-analysis	Studies	Sample Size (*N*)	Breast Cup Size	Most Used Flap	Follow-Up	Complication Rate	Re-Excisions (CtM)	LRR	Distant Recurrence	Oncological Outcomes	Key Notes
Yadav et al. [5]	2026	No	52 retrospective (31/52) prospective 21/52)	2951	NR in all studies	LICAP	Median 23–60 months (varied grossly)	Low Most commonly reported was seroma (5%)	Margin positivity was reported in 19 studies Overall rate 9%	2% Only in 6 studies	4.3%	5-year survival rate >90% DFS only in 3 studies mentioned OS in 5 studies	Only 7/52 used validated PROMs
Pujji et al. [18]	2021	No	11 (8 case series, 2 prospective, 1 RCT)	432	A-D	LICAP (51.9%)	Median 21 months (1–49)	12.3% Most commonly reported was fat necrosis (2.4%)	9.3% (1.3% CtM)	0–0.9% (0.2%)	1.4%		
Nava et al. [2]	2025	Yes	17	1893	A-E	LICAP	Mean 19 months (6–54)	13%	12.9% (1.46% CtM)	NR	NR	NR	- 2 studies report need for symmetrization procedure - 7/17 report patient satisfaction

CtM, completion to mastectomy; LRR, local recurrence rate; LICAP, lateral intercostal artery perforator; NR, not reported; DFS, disease free survival; OS, overall survival; PROMs, patient-reported outcome measures; RCT, randomized control trial.

**Table 3:** Baseline characteristics; patient, tumor and treatment characteristics (*N* = 81).

Patient Characteristics	
Age	57 (37–74)
Cup size A B C D	14 (17.3%) 42 (51.8%) 20 (24.7%) 5 (6.2%)
Tumor characteristics	
Tumor size Type of cancer DCIS DCIS-Mi Invasive carcinoma Multifocal Disease Upper outer quadrant Lower outer quadrant	31 mm (16–55 mm) 14 (17.3%) 2/14 (14.3%) 67 (82.7%) 24/67 (35.8%) 17/24 (70.8%) 7/24 (29.2%)
Treatment characteristics	
NAT (TNBC/HER2+)	11/81 (13.6%)

Variables are presented as median (range), or frequency (*N*%). DCIS, ductal carcinoma in situ; DCIS-Mi, DCIS-microinvasion; NAT, neoadjuvant therapy; TNBC, triple-negative breast cancer.

**Table 4:** Flap characteristics (*N* = 81).

Total CWPF Procedures	81
Flat types used	
LICAP LICAP modifications Meybodi modification Crescent modification LTAP AICAP MICAP	38 (46.9%) 17/38 (44.7%) 4/38 (10.5%) 20 (24.7%) 18 (22.2%) 5 (6.2%)

LICAP, lateral intercostal artery perforator; LTAP, lateral thoracic artery perforator; AICAP, anterior intercostal artery perforator; MICAP, medial intercostal artery perforator.

**Table 5:** Oncological outcomes and postoperative complications (*N* = 81).

Oncological Adequacy	
Margin Status Re-excision rate LRR	Negative No re-excision required 1 case (2 years post-op) Reoperation—Mastectomy due to RT limitation
Postoperative Complication Rate (30 days)	3/81 (3.7%)
Seroma Hematoma SSI Wound-related complication Flap necrosis Lymphoedema Donor-site morbidity	1 (1.2%) (Reoperation—salvage) 2 (2.5%) 1 (1.2%) (Reoperation—LD flap)
Fat Necrosis	6/81 (7.4%) (during follow-up)

LRR, local recurrence rate; RT, radiotherapy; SSI, surgical site infection; LD flap, latissimus dorsi flap.

**Table 6:** Perioperative and postoperative characteristics (*N* = 81).

Operative Flap Harvest Time	18 Min (13–25)
Drain Usage	51 (63%)
Hospital stay duration	
<12 h 12–16 h ~48 h	30 (37%) 49 (60.5%) (with drain usage) 2 (2.5%) (reoperation due to hematoma and flap necrosis respectively)

**Table 7:** The current European landscape.

Region	CWPF Uptake (Approximate Pattern)
UK/Northern Europe	Relatively higher uptake
Western Europe	Moderate and increasing uptake
Southern Europe	Variable and often limited uptake
Eastern/Southeastern Europe	Lower reported uptake

## Data Availability

All original data presented in this study are included in the main text. Further inquiries can be directed to the corresponding author on reasonable request.

## Abbreviations

The following abbreviations are used in this manuscript:

AICAP	anterior intercostal artery perforator
ALND	axillary lymph node dissection
BCS	breast-conserving surgery
CWPF	chest-wall perforator flap
DCIS	ductal carcinoma in situ
DIEP	deep inferior epigastric perforator
IBR	implant-based reconstruction
ICAP	intercostal artery perforator
LD	latissimus dorsi
LICAP	lateral intercostal artery perforator
LTAP	lateral thoracic artery perforator
MICAP	medial intercostal artery perforator
NAT	neoadjuvant therapy
OBS	oncoplastic breast surgery
PBR	partial breast reconstruction
PROMs	patient-reported outcome measures
QoL	quality of life
RCT	randomized controlled trial
RT	radiotherapy
SDC 1	structured data capture 1
SLNB	sentinel lymph node biopsy
SSI	surgical site infection
TDAP	thoracodorsal artery perforator
TNBC	triple-negative breast cancer
TRAM	transverse rectus abdominis myocutaneous
WLE	wide local excision
